# Significances of miRNAs for predicting sepsis mortality: a meta-analysis

**DOI:** 10.3389/fmicb.2025.1472124

**Published:** 2025-03-11

**Authors:** Yuxi Jin, Yue Zhang, Yifei Li, Xiaolan Zheng

**Affiliations:** Key Laboratory of Birth Defects and Related Diseases of Women and Children of MOE, Department of Pediatrics, West China Second University Hospital, Sichuan University, Chengdu, China

**Keywords:** sepsis, miRNA, mortality, prognosis, meta-analysis

## Abstract

**Background:**

Sepsis is a life-threatening condition caused by a dysregulated immune response to infection and remains a major cause of mortality in intensive care units (ICUs). Recent studies have identified microRNAs (miRNAs), a class of small RNA molecules, as potential biomarkers for diagnosing and predicting outcomes in sepsis patients. However, the results of these studies have been inconsistent. This meta-analysis aims to comprehensively evaluate the diagnostic and prognostic value of miRNAs in predicting sepsis-related mortality.

**Methods:**

A comprehensive literature search was performed across major databases, including PubMed, Cochrane Library, EMBASE, and CNKI, up to April 7, 2024. Data extraction and meta-analysis were conducted using Meta-disk 1.4 and STATA 15.1, employing both fixed- and random-effects models to ensure robust statistical analysis.

**Results:**

A total of 55 studies met the inclusion criteria and were analyzed. The pooled sensitivity, specificity, and area under the summary receiver operating characteristic (SROC) curve for miRNA detection were calculated. The overall performance of total miRNA detection demonstrated a sensitivity of 0.76 (95% confidence interval [CI]: 0.74–0.77), a specificity of 0.72 (95% CI: 0.71–0.73), and an SROC value of 0.83. Subgroup analyses revealed that miR-133a-3p exhibited the highest diagnostic accuracy, with a pooled sensitivity of 0.83 (95% CI: 0.70–0.92), specificity of 0.79 (95% CI: 0.71–0.86), and an SROC value of 0.90. Additionally, other miRNAs, including miR-146a, miR-21, miR-210, miR-223-3p, miR-155, miR-25, miR-122, miR-125a, miR-125b, and miR-150, also demonstrated high SROC values (0.84 to 0.76).

**Conclusion:**

This meta-analysis underscores the potential of several microRNAs (miRNAs) as reliable biomarkers for predicting sepsis mortality. Specifically, miR-133a-3p, miR-146a, miR-21, miR-210, miR-223-3p, miR-155, miR-25, miR-122, miR-125b, and miR-150 emerge as promising candidates for clinical applications in sepsis prognosis.

## Introduction

Sepsis is a leading cause of mortality in Intensive Care Units (ICUs) (Venkatesh et al., 2018). It is characterized by a dysregulated immune response to infection that often results in organ failure and high mortality (Singer et al., 2016). Early identification of patients at high risk for sepsis is therefore vital for reducing mortality and improving outcomes (Yang et al., 2022). Current diagnostic and management strategies rely on clinical assessments, monitoring of vital signs, and laboratory parameters, supported by scoring systems such as the Quick Sequential Organ Failure Assessment Score (qSOFA) and the National Early Warning Score (NEWS) (Zhang et al., 2021). Despite their utility, these systems have inherent limitations, necessitating continuous clinical monitoring and highlighting the urgent need for novel predictive indicators of sepsis mortality.

MicroRNAs (miRNAs), a class of small non-coding RNA molecules, have gained attention for their significant role in the regulation of gene expression and immune responses in sepsis (Salim et al., 2020; Sankar et al., 2022; Wang and Han, 2021; Wang et al., 2015). A recent meta-analysis validated the diagnostic utility of miRNAs and identified miR-223-3p as a potential biomarker for sepsis (Shen et al., 2020). However, the predictive value of miRNAs in sepsis mortality remains controversial due to inconsistent findings across studies. To address this gap, this meta-analysis was conducted to systematically evaluate the potential of miRNAs as predictors of sepsis mortality.

## Materials and methods

### Study protocol

This meta-analysis followed a predefined protocol, as recommended by Deeks (2001). The Preferred Reporting Items for Systematic Reviews and Meta-Analyses (PRISMA) Statement was followed for data collection and analysis (Supplementary Table S1) (Moher et al., 2009). The protocol was prospectively registered in PROSPERO (CRD42024530167).

### Search strategy

A systematic search was conducted in the following databases: PubMed, EMBASE, CNKI, and the Cochrane Central Register of Controlled Trials. The search was conducted up to April 7, 2024. The search terms used were (“sepsis” OR “septicemia” OR “pyemia”) AND (“MicroRNAs” OR “MicroRNA” OR “miRNAs” OR “miRNA”). For the PubMed database, the search query was: (Sepsis[MeSH Terms] OR septicemia OR pyemia) AND (MicroRNAs[MeSH Terms] OR MicroRNA OR miRNAs OR miRNA). Detailed search strategies for EMBASE, Cochrane, and CNKI are provided in the Supplementary material. Only studies published in English or Chinese were considered.

### Study selection

The initial step involved the screening of titles and abstracts. Full texts of potentially relevant studies were then retrieved to assess inclusion/exclusion criteria.

The inclusion criteria were as follows: (1) all sepsis patients were confirmed by diagnosis criteria; (2) trials evaluating the expression of microRNAs were included; (3) the data of receiver operating characteristic (ROC) curve and the essential sample size were contained; (4) all reports had specific numbers of sepsis patients who died and survived; and (5) the full text was published in English or Chinese.

The following criteria were used to exclude studies: (1) reviews, letters, conference articles, or case reports; (2) inadequate data for analysis; and (3) duplicated studies.

### Data collection and assessment of study quality

Two independent investigators (Yuxi Jin and Yue Zhang) screened titles and abstracts, with discrepancies resolved by a third reviewer (Yifei Li). Study quality was assessed using the QUADAS-2 checklist (Whiting et al., 2011). Any discrepancies in quality assessments were discussed with the third reviewer. Subsequently, data extraction for sensitivity, specificity, and the number of true positives (TP), false positives (FP), false negatives (FN), and true negatives (TN) was performed by Xiaolan Zheng and Yuxi Jin.

### Evaluation indicators

Sensitivity, specificity, diagnostic odds ratio (DOR), and area under the summary ROC (SROC) curve were calculated. The SROC curve was constructed using sensitivity and specificity, with the area under the curve (AUC) serving as a measure of global performance (Moses et al., 1993).

### Publication bias

Publication bias was quantitatively evaluated using STATA version 15.1, based on funnel plots and Deeks’ test (StataCorp, Texas, United States). In the event that publication bias was detected (*p* < 0.05) (Deeks et al., 2005), the trim-and-fill method was employed to assess its influence. Consistency of results before and after the trim-and-fill adjustment was interpreted as evidence of stability and reliability (Lin and Chu, 2018).

### Heterogeneity and meta-regression

Heterogeneity was assessed using the Chi-squared (χ^2^) test for pooled sensitivity and specificity, and the Cochran Q test for pooled DOR. Statistical heterogeneity was defined as *p* < 0.05. The *I*^2^ test was also employed to measure the proportion of variability across studies, with values of 25, 50, and 75% representing low, moderate, and high heterogeneity, respectively (Higgins and Thompson, 2002). When heterogeneity was detected, meta-regression analyses were performed using STATA 15.1 to explore potential sources, such as sample type and population differences. A *p* value < 0.05 was considered significant.

### Sensitivity analysis and subgroup analysis

Sensitivity analysis was performed using STATA 15.1 to evaluate the influence of individual studies on overall results. Subgroup analyses were performed using Meta-Disc 1.4.

### Statistical analysis

Meta-Disc 1.4 was used for data processing and for the analysis of threshold effects. Publication bias was assessed using STATA 15.1. For homogeneous results, a fixed-effects model was employed, while for heterogeneous results (*I*^2^ > 50%), a random-effects model was applied. The generation of forest plots was instrumental in the visualization of the results.

## Results

### Search results

**Figure 1:**
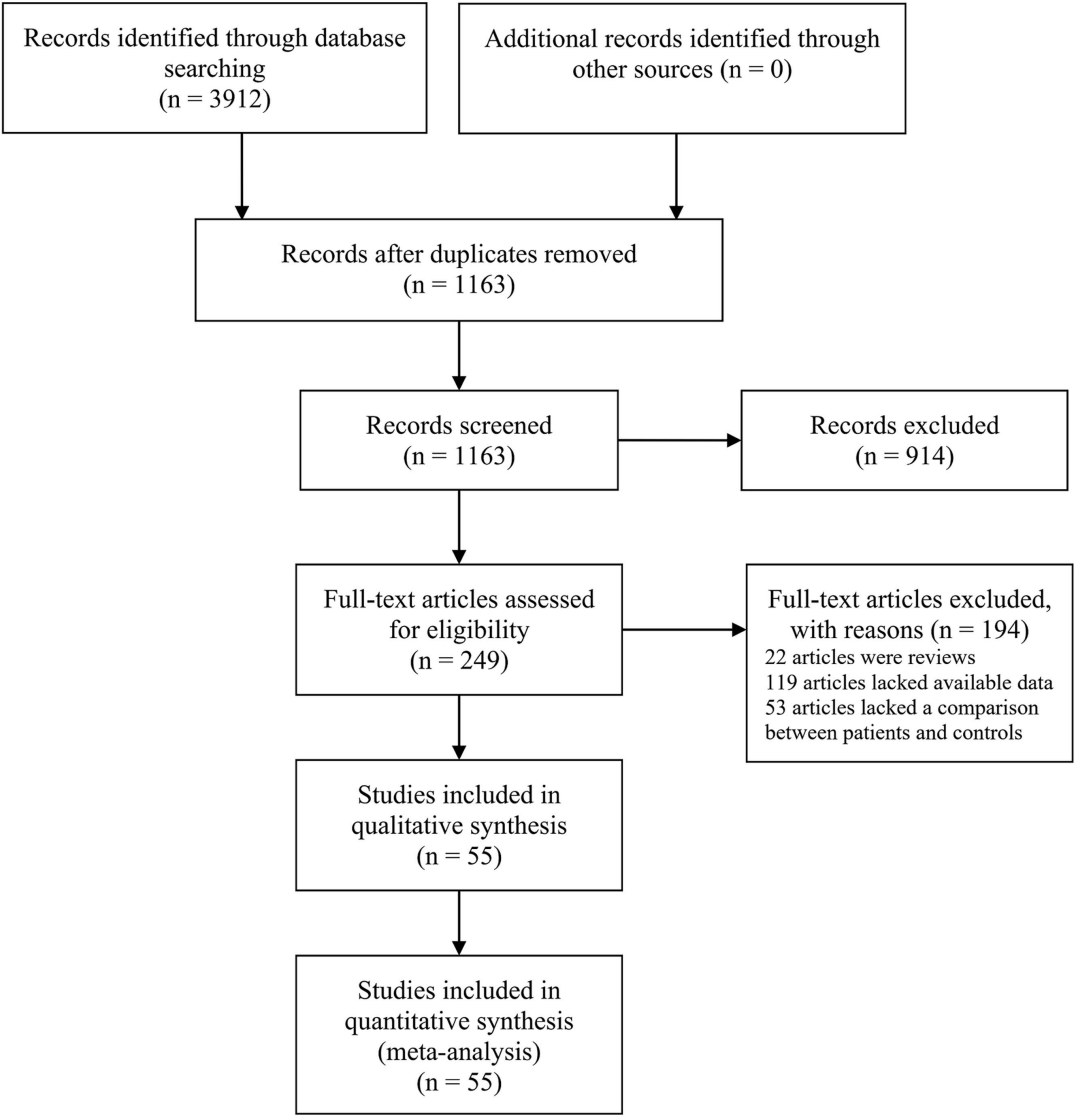
Flow diagram of the study selection process.

The initial search yielded 3,912 papers, of which 249 articles were deemed suitable for full-text review following an initial assessment of their titles and abstracts. However, further evaluation revealed that 22 articles were excluded due to inappropriate article types, and 119 studies lacked data on TP, FP, FN, and TN cases. Additionally, 53 articles did not include a comparison between survival and non-survival sepsis patients. The study selection process is detailed in Figure 1. Ultimately, 55 studies were included in the meta-analysis (Yang et al., 2022; Salim et al., 2020; Sankar et al., 2022; Wang et al., 2012; Yang et al., 2015; Yao et al., 2015; Yang et al., 2016; Huo et al., 2017; Lin, 2017; Rahmel et al., 2018; Zhang, 2018; Zhang et al., 2018; Lin et al., 2019; Sun et al., 2019; Xue et al., 2019; Chen L. et al., 2020; Chen W. et al., 2020; Dou et al., 2020; Lin et al., 2020; Liu et al., 2020; Na et al., 2020; Qiu et al., 2020; Wang H. et al., 2020; Wang J. et al., 2020; Wang L. et al., 2020; Wang Q. et al., 2020; Xu et al., 2020; Yang J. et al., 2020; Yang Y. et al., 2020; Zhao et al., 2020; Zhu, 2020; Deng et al., 2021; Guo et al., 2021; Ma et al., 2021; Pan et al., 2021; Wang H. et al., 2021; Xu et al., 2021; Yang et al., 2021; Yao et al., 2021; Deng et al., 2022; Li et al., 2022; Pan et al., 2022; Yu et al., 2022; Zhang et al., 2022; Zhao et al., 2022; Ali et al., 2023; Bao and Zhang, 2023; Bian and Pang, 2023; Li N. et al., 2023; Liu and Yang, 2023; Yao et al., 2023; Zhang et al., 2023; Behroozizad et al., 2024; Hao et al., 2024; Zhang et al., 2024), encompassing a total of 6,443 sepsis patients, of whom 2,047 were non-survivors and 4,396 were survivors. These studies analyzed the roles of 41 different microRNAs (miRNAs), 11 of which (miR-133a-3p, miR-146a, miR-21, miR-210, miR-223-3p, miR-155, miR-25, miR-122, miR-125a, miR-125b, and miR-150) were examined in more than two investigations. Full-text articles published in both English and Chinese were included. Of the 55 studies analyzed, 27 (Yang et al., 2015; Yang et al., 2016; Huo et al., 2017; Zhang, 2018; Zhang et al., 2018; Sun et al., 2019; Xue et al., 2019; Qiu et al., 2020; Wang H. et al., 2020; Wang L. et al., 2020; Xu et al., 2020; Yang J. et al., 2020; Pan et al., 2021; Yang et al., 2021; Deng et al., 2022; Li et al., 2022; Pan et al., 2022; Yu et al., 2022; Zhang et al., 2022; Zhao et al., 2022; Bao and Zhang, 2023; Bian and Pang, 2023; Li N. et al., 2023; Liu and Yang, 2023; Yao et al., 2023; Zhang et al., 2023; Zhang et al., 2024) were published in Chinese, ensuring a comprehensive review of data from both English and Chinese sources. The patient population spanned a range of ages, with four studies focusing on neonates (less than 28 days old), five on children older than one month, and 46 on adults. Sample types included plasma (26 studies), serum (25 studies), peripheral blood mononuclear cells (PBMCs) (2 studies), and whole blood (2 studies). The geographical distribution of the studies was as follows: 52 studies were conducted in Asia (50 in China, 1 in India, and 1 in Iran), 2 in Africa (Egypt), and 1 in Europe (Germany). Among the 55 studies, 30 had a sample size of ≥100, while 25 included fewer than 100 participants. Diagnostic criteria for sepsis varied significantly, with two studies adhering to Sepsis-1.0, 13 studies using Sepsis-2.0, and 38 studies adopting Sepsis-3.0 criteria. Two studies did not specify the criteria utilized. All included studies employed quantitative reverse transcription PCR (qRT-PCR) for miRNA detection, ensuring high sensitivity and specificity in the quantification of these molecules across different sample types. Regarding reference genes for qRT-PCR, 41 studies used U6, 8 employed non-U6 genes, and 6 did not specify the reference gene. Table 1 summarizes the characteristics of the included studies.

**Table 1:** Characteristics of studies in meta-analysis.

No.	First author	Year	Country	Population	Follow-up time	Golden standard	Reference genes of qRT-PCR	Specimen	Death (male/female)	Survival (male/female)	Selected miRNAs
1	Hao J	2024	China	Adults	Sepsis 3.0	28 days	U6	Plasma	53 (33/20)	112 (64/48)	miR-210, miR-494
2	Behroozizad N	2024	Iran	Adults	Sepsis 1.0	N/R	GAPDH	Plasma	29 (N/R)	71 (N/R)	miR-135a, miR-193
3	Zhang R	2024	China	Adults	Sepsis 2.0	28 days	U6	Whole blood	21 (N/R)	82 (N/R)	miR-125b, miR-150
4	Liu W	2023	China	Adults	Sepsis 3.0	28 days	U6	Serum	19 (10/9)	54 (19/35)	miR-130a
5	Bao Z	2023	China	Adults	Sepsis 3.0	28 days	U6	Serum	30 (17/13)	107 (55/52)	miR-451, miR-223
6	Zhang Y	2023	China	Adults	Sepsis 3.0	28 days	U6	Serum	54 (N/R)	103 (N/R)	miR-92a-3p, miR-147
7	Yao Y	2023	China	Adults	Sepsis 3.0	28 days	U6	PBMCs	21 (N/R)	61 (N/R)	miR-122, miR-146a
8	Li N	2023	China	Adults	Sepsis 3.0	28 days	N/R	Serum	52 (30/22)	77 (43/34)	miR-98-5p
9	Bian M	2023	China	Adults	Sepsis 3.0	28 days	U6	Whole blood	14 (N/R)	31 (N/R)	miR-16, miR-25, miR-92a-3p, miR-103, miR-107
10	Ali MA	2023	Egypt	Neonates	N/R	20 days	miR-16	Serum	22 (N/R)	38 (N/R)	miR-181b-5p, miR-21
11	Zhang C	2022	China	Neonates	Sepsis 2.0	28 days	U6	Plasma	12 (N/R)	53 (N/R)	miR-96
12	Yu L	2022	China	Adults	Sepsis 2.0	28 days	U6	Serum	43 (N/R)	115 (N/R)	miR-206, miR-451
13	Deng Y	2022	China	Children	Sepsis 3.0	28 days	U6	Plasma	34 (N/R)	74 (N/R)	miR-455-5p, miR-483-5p
14	Pan Y	2022	China	Adults	Sepsis 3.0	28 days	U6	Plasma	51 (28/23)	33 (19/14)	miR-223-3p, miR-124a
15	Zhao Y	2022	China	Adults	Sepsis 3.0	28 days	U6	Serum	14 (N/R)	21 (N/R)	miR-133a-3p
16	Li C	2022	China	Adults	Sepsis 3.0	28 days	U6	Serum	19 (N/R)	61 (N/R)	miR-122, miR-133a-3p
17	Yang J	2022	China	Adults	Sepsis 3.0	28 days	U6	Plasma	138 (91/47)	299 (182/117)	miR-150
18	Sankar S	2022	India	Neonates	N/R	28 days	SNORD61	Plasma	14 (N/R)	28 (N/R)	miR-146a
19	Yang X	2021	China	Adults	Sepsis 3.0	28 days	U6	Serum	29 (16/13)	73 (39/34)	miR-10a-5p, miR-21
20	Wang H	2021	China	Adults	Sepsis 3.0	28 days	U6	Plasma	45 (N/R)	101 (N/R)	miR-223-3p
21	Pan Q	2021	China	Adults	Sepsis 3.0	28 days	N/R	Serum	68 (36/32)	192 (98/94)	miR-150, miR-146a
22	Ma W	2021	China	Children	Sepsis 2.0	28 days	U6	Serum	48 (28/20)	32 (16/16)	miR-122
23	Deng Y	2021	China	Children	Sepsis 2.0	28 days	U6	Plasma	46 (N/R)	107 (N/R)	miR-101-3p, miR-141-3p
24	Yao J	2021	China	Adults	Sepsis 3.0	28 days	U6	Plasma	70 (53/17)	81 (60/21)	miR-519c-5p
25	Xu C	2021	China	Adults	Sepsis 3.0	28 days	U6	Serum	33 (N/R)	36 (N/R)	miR-21, miR-210
26	Guo W	2021	China	Adults	Sepsis 3.0	28 days	U6	Serum	17 (N/R)	103 (N/R)	miR-29a
27	Xu Z	2020	China	Adults	Sepsis 3.0	28 days	U6	Serum	37 (N/R)	63 (N/R)	miR-125b, miR-142-3p
28	Yang J	2020	China	Children	Sepsis 2.0	28 days	U6	plasma	27 (N/R)	50 (N/R)	miR-146a, miR-223-3p
29	Wang L	2020	China	Adults	Sepsis 3.0	23 days	U6	Serum	7 (N/R)	48 (N/R)	miR-205
30	Wang J	2020	China	Adults	Sepsis 3.0	23 days	N/R	Serum	38 (20/18)	44 (23/21)	miR-25
31	Qiu Y	2020	China	Adults	Sepsis 3.0	28 days	U6	Serum	57 (N/R)	105 (N/R)	miR-146a
32	Zhu X	2020	China	Adults	Sepsis 2.0	28 days	U6	plasma	39 (N/R)	81 (N/R)	miR-125a, miR-125b
33	Zhao D	2020	China	Adults	Sepsis 3.0	28 days	U6	plasma	41 (N/R)	109 (N/R)	miR-125a, miR-125b
34	Yang Y	2020	China	Adults	Sepsis 3.0	28 days	U6	plasma	24 (N/R)	78 (N/R)	miR-125a
35	Wang Q	2020	China	Adults	Sepsis 3.0	28 days	U6	plasma	62 (N/R)	134 (N/R)	miR-103, miR-107
36	Wang H	2020	China	Adults	Sepsis 2.0	N/R	U6	plasma	36 (N/R)	96 (N/R)	miR-146a
37	Salim RF	2020	Egypt	Neonates	Sepsis 2.0	28 days	U6	Serum	16 (N/R)	34 (N/R)	miR-21
38	Na L	2020	China	Adults	Sepsis 3.0	28 days	U6	Plasma	56 (N/R)	163 (N/R)	miR-21
39	Liu W	2020	China	Adults	Sepsis 3.0	28 days	U6	Plasma	56 (N/R)	140 (N/R)	miR-125a
40	Lin R	2020	China	Adults	Sepsis 3.0	28 days	U6	Plasma	69 (N/R)	139 (N/R)	miR-126
41	Dou H	2020	China	Adults	Sepsis 2.0	28 days	U6	Serum	55 (N/R)	148 (N/R)	miR-155, miR-143
42	Chen W	2020	China	Adults	Sepsis 3.0	28 days	U6	Plasma	26 (N/R)	78 (N/R)	miR-146b
43	Chen L	2020	China	Adults	Sepsis 3.0	28 days	U6	Plasma	48 (N/R)	132 (N/R)	miR-146a, miR-146b
44	Xue Y	2019	China	Adults	Sepsis 3.0	28 days	N/R	Serum	19 (N/R)	35 (N/R)	miR-133a-3p
45	Sun Z	2019	China	Adults	Sepsis 3.0	28 days	N/R	Plasma	21 (14/7)	21 (12/9)	miR-130a
46	Lin Y	2019	China	Adults	Sepsis 1.0	28 days	cel-miR-39-3p	Plasma	24 (N/R)	68 (N/R)	miR-210, miR-494, miR-205
47	Zhang H	2018	China	Adults	Sepsis 2.0	3 months	U6	Serum	28 (N/R)	54 (N/R)	miR-155
48	Zhang C	2018	China	Children	Sepsis 3.0	N/R	U6	PBMCs	25 (15/10)	62 (43/19)	miR-132
49	Rahmel T	2018	Germany	Adults	Sepsis 3.0	30 days	cel-miR-54	Serum	40 (25/15)	68 (39/29)	miR-122
50	Lin H	2017	China	Adults	Sepsis 3.0	28 days	cel-miR-39-3p	plasma	35 (N/R)	47 (N/R)	miR-210
51	Huo R	2017	China	Adults	Sepsis 3.0	28 days	N/R	Serum	21 (N/R)	53 (N/R)	miR-29a, miR-10a-5p
52	Yang W	2016	China	Adults	Sepsis 3.0	28 days	U6	Plasma	21 (N/R)	40 (N/R)	miR-155
53	Yao L	2015	China	Adults	Sepsis 2.0	28 days	miR-16	Serum	25 (N/R)	45 (N/R)	miR-25
54	Yang W	2015	China	Adults	Sepsis 3.0	28 days	miR-16	Plasma	25 (N/R)	23 (N/R)	miR-150
55	Wang H	2012	China	Adults	Sepsis 2.0	28 days	U6	Serum	73 (N/R)	93 (N/R)	miR-223-3p, miR-15a, miR-16, miR-122, miR-193b, miR-483-5p

miR, mircoRNA; PBMCs, peripheral blood mononuclear cells; N/R, not report.

### Study quality

The quality of the included studies was assessed using the QUADAS-2 tool, which indicated a high risk of bias for the index test (Supplementary Figure S1).

### Predicting accuracy of miRNAs

#### Total mixed miRNAs

**Figure 2:**
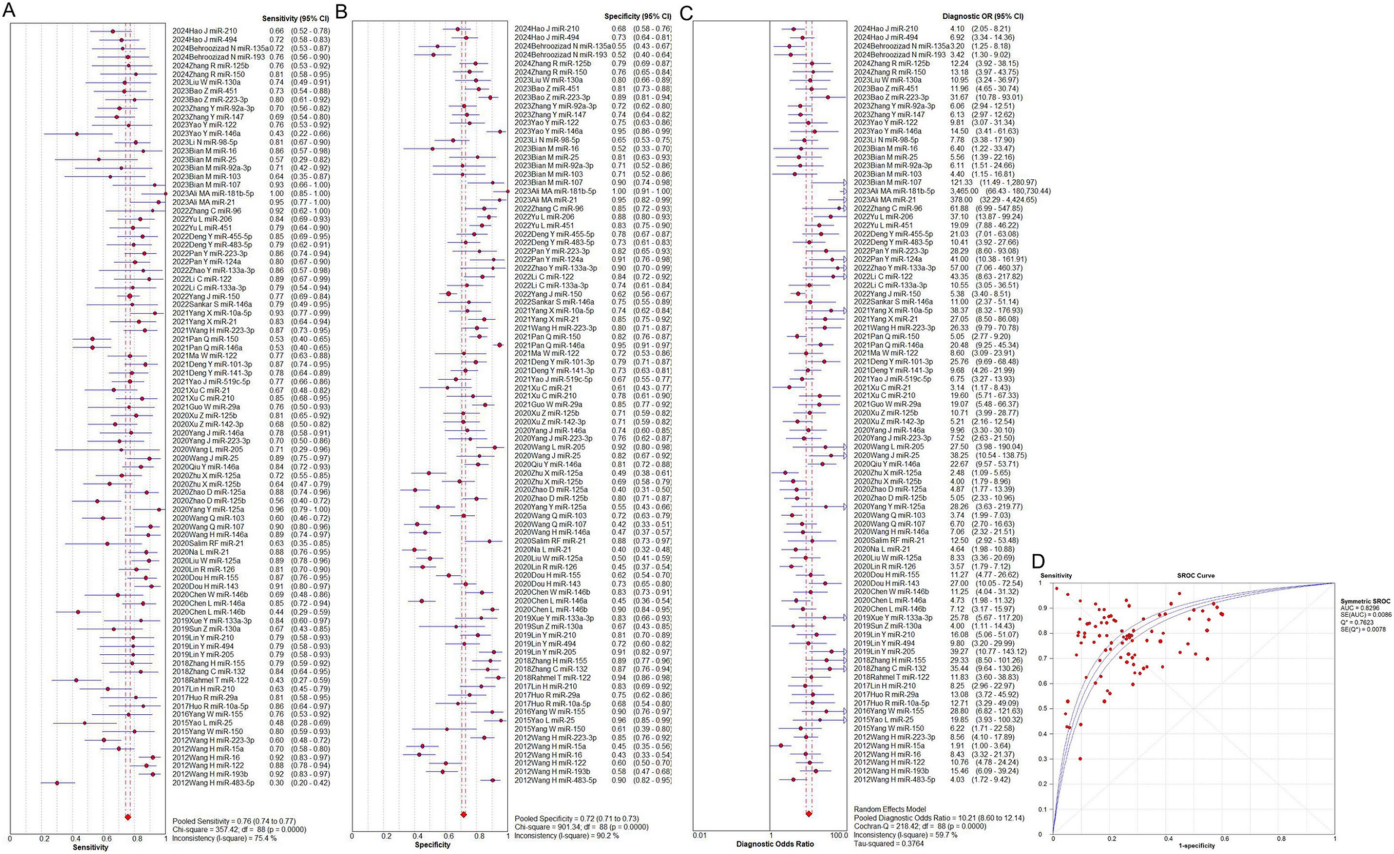
Performance of total miRNAs detection for sepsis diagnosis. **(A)** Pooled sensitivity. **(B)** Pooled specificity. **(C)** Overall DOR. **(D)** The SROCs for all datasets. CI, confidence interval; DOR, predicting odds ratio; miR, mircoRNA; OR, odds ratio; SROC, summary receiver operating characteristic curves value.

The overall predictive performance of total mixed microRNAs (T miR) for identifying sepsis is shown in Figure 2. The pooled sensitivity was 0.76 (95% CI: 0.74–0.77), accompanied by significant heterogeneity (*p* < 0.0001, χ^2^ = 357.42, *I*^2^ = 75.4%, Figure 2A). The pooled specificity was 0.72 (95% CI: 0.71–0.73), also exhibiting notable heterogeneity (*p* < 0.0001, χ^2^ = 901.34, *I*^2^ = 90.2%, Figure 2B). The pooled diagnostic odds ratio (DOR) was 10.21 (95% CI: 8.60–12.14), with substantial heterogeneity (*p* < 0.0001, Cochran Q = 218.42, *I*^2^ = 59.7%, Figure 2C). The area under the curve (AUC) value was 0.83 (Figure 2D). The absence of a curvilinear form in the SROC curve indicates that no threshold effect was detected.

**Figure 3:**
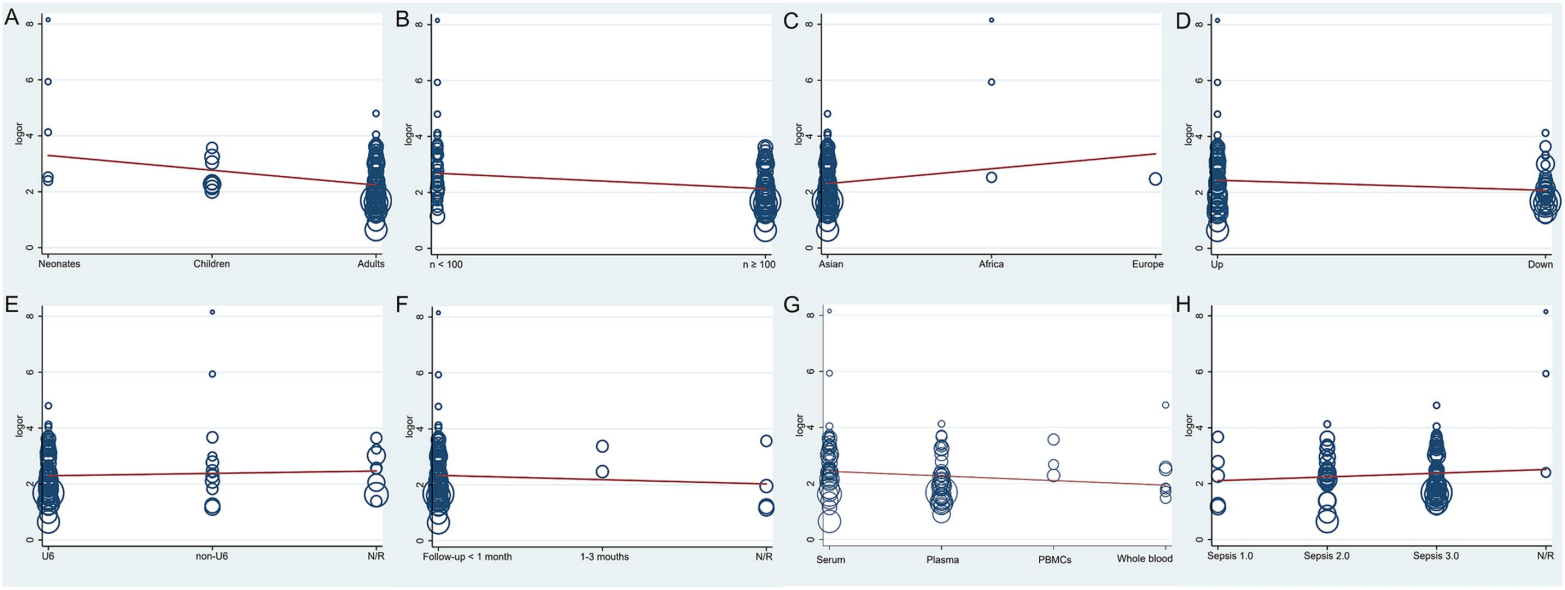
The meta-regression of the enrolled studies. **(A)** For the population, the meta-regression found a significant impact on the homogeneity of the included studies, *p* = 0.013, *t* = −2.53, 95%CI 0.39–0.89. **(B)** For the total sample size, the meta-regression found it was a dramatic impact on the homogeneity of the enrolled studies, *p* = 0.003, *t* = −3.01, 95%CI 0.40–0.83. **(C)** For the region, the meta-regression did not detect it had a dramatic impact on the homogeneity of the enrolled studies, *p* = 0.174, *t* = 1.37, 95%CI 0.79–3.68. **(D)** For the miRNA expression level, the meta-regression did not detect it was a dramatic impact on the homogeneity of the enrolled studies, *p* = 0.06, *t* = −1.90, 95%CI 0.48–1.02. **(E)** For the qRT-PCR reference genes, the meta-regression did not find it was a dramatic impact on the homogeneity of the enrolled studies, *p* = 0.565, *t* = 0.58, 95%CI 0.81–1.46. **(F)** For the follow-up time, the meta-regression did not detect it was a dramatic impact on the homogeneity of the included studies, *p* = 0.462, *t* = −0.74, 95%CI 0.57–1.30. **(G)** For the type of samples, the meta-regression did not detect it was a dramatic impact on the homogeneity of the enrolled studies, *p* = 0.154, *t* = −1.44, 95%CI 0.68–1.06. **(H)** For the sepsis diagnostic criteria, the meta-regression did not detect it was a dramatic impact on the homogeneity of the enrolled studies, *p* = 0.372, *t* = 0.90, 95%CI 0.85–1.53.

To identify potential sources of heterogeneity, a meta-regression analysis was conducted, evaluating factors such as sample type, geographical location, sepsis diagnostic criteria, reference genes for qRT-PCR, patient age, follow-up duration, miRNA expression levels (upregulated or downregulated), and total sample size. Population (*p* = 0.013, *t* = −2.53, 95% CI: 0.39–0.89, Figure 3A) and total sample size (*p* = 0.003, *t* = −3.01, 95% CI: 0.40–0.83, Figure 3B) were identified as significant contributors to heterogeneity, whereas the remaining factors were not significant (*p* > 0.05, Figures 3C–H).

#### Subgroup analysis

Subgroup analysis was performed based on population and sample size to investigate the sources of heterogeneity. Furthermore, a comprehensive analysis of 11 specific microRNAs (namely, miR-133a-3p, miR-146a, miR-21, miR-210, miR-223-3p, miR-155, miR-25, miR-122, miR-125a, miR-125b, and miR-150) was conducted in studies that have examined them in more than two investigations. The results of these investigations are presented in Table 2 and Supplementary Figures S2–S14.

**Table 2:** Subgroup analysis results of included studies.

	Sensitivity (95% CI)	Specificity (95% CI)	DOR (95% CI)	SROC (AUC ± SE)
**Population**				
Neonates	0.87 (0.78–0.93)	0.89 (0.84–0.93)	60.89 (10.99–337.27)	0.96 ± 0.04
P/I^2^	0.0046/73.4%	0.0037/74.3%	0.0094/70.2%	–
Children	0.80 (0.75–0.85)	0.77 (0.73–0.80)	13.02 (9.28–18.27)	0.84 ± 0.02
P/I^2^	0.7472/0%	0.4442/0%	0.3805/6.4%	–
Adults	0.75 (0.73–0.76)	0.71 (0.70–0.72)	9.49 (7.93–11.36)	0.82 ± 0.01
P/I^2^	<0.0001/77.1%	<0.0001/91.0%	<0.0001/59.1%	–
**Type of sample size**				
*n* < 100	0.78 (0.75–0.80)	0.81 (0.79–0.83)	14.18 (11.61–17.30)	0.86 ± 0.01
P/I^2^	0.0008/47.5%	<0.0001/67.9%	0.0048/41.3%	–
*n* ≥ 100	0.75 (0.73–0.77)	0.69 (0.68–0.70)	8.42 (6.90–10.28)	0.81 ± 0.01
P/I^2^	<0.0001/82.4%	<0.0001/92.7%	<0.0001/62.5%	–
**Type of miRNAs**				
miR-133a-3p	0.83 (0.70–0.92)	0.79 (0.71–0.86)	18.87 (8.19–43.48)	0.90 ± 0.03
P/I^2^	0.8599/0%	0.1964/38.6%	0.3531/3.9%	–
miR-146a	0.73 (0.67–0.78)	0.73 (0.70–0.77)	11.14 (7.63–16.28)	0.84 ± 0.02
P/I^2^	<0.0001/83.0%	<0.0001/96.3%	0.1539/36.0%	–
miR-21	0.81 (0.74–0.87)	0.63 (0.57–0.68)	13.13 (3.94–43.80)	0.87 ± 0.07
P/I^2^	0.0136/68.2%	<0.0001/95.3%	0.0007/79.1%	–
miR-210	0.72 (0.64–0.79)	0.75 (0.70–0.80)	9.09 (4.24–19.47)	0.83 ± 0.05
P/I^2^	0.1132/49.7%	0.1074/50.7%	0.0739/56.8%	–
miR-223-3p	0.75 (0.69–0.81)	0.83 (0.79–0.87)	16.24 (8.65–30.49)	0.89 ± 0.02
P/I^2^	0.0030/75.0%	0.2676/23.0%	0.0892/50.4%	–
miR-155	0.83 (0.74–0.89)	0.73 (0.67–0.78)	16.34 (8.76–30.46)	0.89 ± 0.02
P/I^2^	0.4159/0%	<0.0001/91.5%	0.3379/7.8%	–
miR-25	0.70 (0.59–0.80)	0.87 (0.79–0.92)	16.31 (5.05–52.68)	0.89 ± 0.07
P/I^2^	0.0007/86.3%	0.0581/64.9%	0.1307/50.8%	–
miR-122	0.75 (0.69–0.81)	0.76 (0.71–0.81)	11.81 (7.44–18.73)	0.84 ± 0.02
P/I^2^	<0.0001/86.2%	<0.0001/86.6%	0.5561/0%	–
miR-125a	0.86 (0.79–0.91)	0.48 (0.43–0.53)	5.69 (2.50–12.95)	0.51 ± 0.12
P/I^2^	0.0369/64.7%	0.2191/32.2%	0.0725/57.0%	–
miR-125b	0.68 (0.60–0.76)	0.76 (0.71–0.80)	6.36 (4.18–9.68)	0.80 ± 0.03
P/I^2^	0.0828/55.1%	0.2584/25.5%	0.2667/24.1%	–
miR-150	0.71 (0.65–0.77)	0.70 (0.66–0.74)	5.75 (4.21–7.87)	0.76 ± 0.02
P/I^2^	0.0024/79.0%	<0.0001/88.0%	0.5495/0%	–

CI, confidence interval; DOR, diagnostic odds ratio; SROC, summary receiver operating characteristic curves value; AUC, area under the curve; SE, standard error.

#### Population

The population was segmented into three distinct categories: neonates, children, and adults. In the neonate population (see Supplementary Figure S2), the pooled sensitivity was 0.87 (95%CI 0.78–0.93), indicating moderate heterogeneity (*p* = 0.0046, χ^2^ = 15.03, *I*^2^ = 73.4%, see Supplementary Figure S2A). Similarly, the pooled specificity was 0.89 (95%CI 0.84–0.93), also demonstrating moderate heterogeneity (*p* = 0.0037, χ^2^ = 15.56, *I*^2^ = 74.3%, see Supplementary Figure S2B). The pooled DOR was 60.89 (95%CI 10.99–337.27), with moderate heterogeneity (*p* = 0.0094, Cochran Q = 13.42, *I*^2^ = 70.2%, Supplementary Figure S2C). The area under the curve (AUC) value was 0.96 ± 0.04 (Supplementary Figure S2D).

For the pediatric population, the pooled sensitivity was 0.80 (95%CI 0.75–0.85), exhibiting no heterogeneity (*p* = 0.7472, χ^2^ = 4.28, *I*^2^ = 0%, Supplementary Figure S2E). The pooled specificity was 0.77 (95%CI 0.73–0.80), with no heterogeneity (*p* = 0.4442, χ^2^ = 6.85, *I*^2^ = 0%, Supplementary Figure S2F). The pooled DOR was 13.02 (95%CI 9.28–18.27), with no heterogeneity (*p* = 0.3805, Cochran Q = 7.48, *I*^2^ = 6.4%, Supplementary Figure S2G). The AUC value was 0.84 ± 0.02 (Supplementary Figure S2H).

In adults, the pooled sensitivity was 0.75 (95%CI 0.73–0.76), with high heterogeneity (*p* < 0.0001, χ^2^ = 326.86, *I*^2^ = 77.1%, Supplementary Figure S2I). The pooled specificity was 0.71 (95%CI 0.70–0.72), also with notable heterogeneity (*p* < 0.0001, χ^2^ = 834.66, *I*^2^ = 91.0%, Supplementary Figure S2J). The pooled DOR was 9.49 (95%CI 7.93–11.36), with moderate heterogeneity (*p* < 0.0001, Cochran Q = 183.54, *I*^2^ = 59.1%, Supplementary Figure S2K). The AUC value was 0.82 ± 0.01 (Supplementary Figure S2L).

#### Sample size

The predictive performance of studies with small sample sizes is shown in Supplementary Figures S3A–D. The pooled sensitivity was 0.78 (95% CI: 0.75–0.80), with low heterogeneity (*p* = 0.0008, χ^2^ = 70.4, *I*^2^ = 47.5%, Supplementary Figure S3A). The pooled specificity was 0.81 (95% CI: 0.79–0.83), with moderate heterogeneity (*p* < 0.0001, χ^2^ = 115.15, *I*^2^ = 67.9%, Supplementary Figure S3B). The pooled DOR was 14.18 (95% CI: 11.61–17.30), with low heterogeneity (*p* = 0.0048, Cochran Q = 63.08, *I*^2^ = 41.3%, Supplementary Figure S3C). The AUC value was 0.86 ± 0.01 (Supplementary Figure S3D).

In studies with large sample sizes, the pooled sensitivity was 0.75 (95% CI: 0.73–0.77), with significant heterogeneity (*p* < 0.0001, χ^2^ = 284.60, *I*^2^ = 82.4%, Supplementary Figure S3E). The pooled specificity was 0.69 (95% CI: 0.68–0.70), with substantial heterogeneity (*p* < 0.0001, χ^2^ = 686.24, *I*^2^ = 92.7%, Supplementary Figure S3F). The pooled DOR was 8.42 (95% CI: 6.90–10.28), with moderate heterogeneity (*p* < 0.0001, Cochran Q = 133.42, *I*^2^ = 62.5%, Supplementary Figure S3G). The AUC value was 0.81 ± 0.01 (Supplementary Figure S3H).

### Specific miRNAs

#### miRNA-133a-3p

Three reports (Xue et al., 2019; Li et al., 2022; Zhao et al., 2022) assessed miR-133a-3p. The pooled sensitivity was 0.83 (95% CI: 0.70–0.92), with no heterogeneity (*p* = 0.8599, χ^2^ = 0.30, *I*^2^ = 0%, Supplementary Figure S4A). The pooled specificity was 0.79 (95% CI: 0.71–0.86), with low heterogeneity (*p* = 0.1964, χ^2^ = 3.26, *I*^2^ = 38.6%, Supplementary Figure S4B). The pooled DOR was 18.87 (95% CI: 8.19–43.48), with no heterogeneity (*p* = 0.3531, Cochran Q = 2.08, *I*^2^ = 3.9%, Supplementary Figure S4C). The AUC was 0.90 ± 0.03 (Supplementary Figure S4D).

#### miRNA-146a

Seven studies (Sankar et al., 2022; Chen L. et al., 2020; Qiu et al., 2020; Wang H. et al., 2020; Yang J. et al., 2020; Pan et al., 2021; Yao et al., 2023) analyzed miR-146a. The pooled sensitivity was 0.73 (95% CI: 0.67–0.78), and specificity was 0.73 (95% CI: 0.70–0.77), both showing significant heterogeneity (sensitivity: *p* < 0.0001, χ^2^ = 35.20, *I*^2^ = 83.0%; specificity: *p* < 0.0001, χ^2^ = 162.24, *I*^2^ = 96.3%, Supplementary Figures S5A,B). The pooled DOR was 11.14 (95% CI: 7.63–16.28), with low heterogeneity (*p* = 0.1539, Cochran Q = 9.37, *I*^2^ = 36.0%, Supplementary Figure S5C). The AUC was 0.84 ± 0.02 (Supplementary Figure S5D).

#### miRNA-21

Five reports (Salim et al., 2020; Na et al., 2020; Xu et al., 2021; Yang et al., 2021; Ali et al., 2023) examined miR-21. The pooled sensitivity was 0.81 (95% CI: 0.74–0.87), with significant heterogeneity (*p* = 0.0136, χ^2^ = 12.56, *I*^2^ = 68.2%, Supplementary Figure S6A). The specificity was 0.63 (95% CI: 0.57–0.68), also showing high heterogeneity (*p* < 0.0001, χ^2^ = 85.61, *I*^2^ = 95.3%, Supplementary Figure S6B). The pooled DOR was 13.13 (95% CI: 3.94–43.80), with high heterogeneity (*p* = 0.0007, Cochran Q = 19.11, *I*^2^ = 79.1%, Supplementary Figure S6C). The AUC was 0.87 ± 0.07 (Supplementary Figure S6D).

#### miRNA-210

Four reports (Lin, 2017; Lin et al., 2019; Xu et al., 2021; Hao et al., 2024) evaluated miR-210. The pooled sensitivity was 0.72 (95% CI: 0.64–0.79), with low heterogeneity (*p* = 0.1132, χ^2^ = 5.97, *I*^2^ = 49.7%, Supplementary Figure S7A). The specificity was 0.75 (95% CI: 0.70–0.80), with moderate heterogeneity (*p* = 0.1074, χ^2^ = 6.09, *I*^2^ = 50.7%, Supplementary Figure S7B). The pooled DOR was 9.09 (95% CI: 4.24–19.47), with moderate heterogeneity (*p* = 0.0739, Cochran Q = 6.94, *I*^2^ = 56.8%, Supplementary Figure S7C). The AUC was 0.83 ± 0.05 (Supplementary Figure S7D).

#### miRNA-223-3p

Five studies (Wang et al., 2012; Yang J. et al., 2020; Wang H. et al., 2021; Pan et al., 2022; Bao and Zhang, 2023) assessed miR-223-3p. The pooled sensitivity was 0.75 (95% CI: 0.69–0.81), with significant heterogeneity (*p* = 0.0030, χ^2^ = 16.00, *I*^2^ = 75.0%, Supplementary Figure S8A). The pooled specificity was 0.83 (95% CI: 0.79–0.87), showing no heterogeneity (*p* = 0.2676, χ^2^ = 5.20, *I*^2^ = 23.0%, Supplementary Figure S8B). The pooled DOR was 16.24 (95% CI: 8.65–30.49), with moderate heterogeneity (*p* = 0.0892, Cochran Q = 8.06, *I*^2^ = 50.4%, Supplementary Figure S8C). The AUC was 0.89 ± 0.02 (Supplementary Figure S8D).

#### miRNA-155

Three studies (Yang et al., 2016; Zhang et al., 2018; Dou et al., 2020) assessed the predictive performance of microRNA-155 (Supplementary Figure S9). The pooled sensitivity was 0.83 (95%CI 0.74–0.89), with no heterogeneity (*p* = 0.4159, χ^2^ = 1.75, *I*^2^ = 0%, Supplementary Figure S9A). The pooled specificity was 0.73 (95%CI 0.67–0.78), with high heterogeneity (*p* < 0.0001, χ^2^ = 23.59, *I*^2^ = 91.5%, Supplementary Figure S9B). The pooled DOR was 16.34 (95%CI 8.76–30.46), with no heterogeneity (*p* = 0.3379, Cochran-Q = 2.17, *I*^2^ = 7.8%, Supplementary Figure S9C). The AUC value was 0.89 ± 0.02 (Supplementary Figure S9D).

#### miRNA-25

The overall predicting performance of miR-25 was analyzed using three studies (Yao et al., 2015; Wang J. et al., 2020; Bian and Pang, 2023) (Supplementary Figure S10). The pooled sensitivity was 0.70 (95%CI 0.59–0.80), and the specificity was 0.87 (95%CI 0.79–0.92). The presence of significant heterogeneity was identified (sensitivity: *p* = 0.0007, χ^2^ = 14.59, *I*^2^ = 86.3%; specificity: *p* = 0.0581, χ^2^ = 5.69, *I*^2^ = 64.9%, Supplementary Figures S10A,B). The pooled DOR was 16.31 (95%CI 5.05–52.68), with moderate heterogeneity (*p* = 0.1307, Cochran-Q = 4.07, *I*^2^ = 50.8%, Supplementary Figure S10C). The AUC value was 0.89 ± 0.07 (Supplementary Figure S10D).

#### miRNA-122

Five studies (Wang et al., 2012; Rahmel et al., 2018; Ma et al., 2021; Li et al., 2022; Yao et al., 2023) assessed the predictive performance of miR-122 (see Supplementary Figure S11). The pooled sensitivity was 0.75 (95% CI 0.69–0.81), and the specificity was 0.76 (95% CI 0.71–0.81). Notably, significant heterogeneity was observed in the sensitivity analysis (sensitivity: *p* < 0.0001, χ^2^ = 28.93, *I*^2^ = 86.2%; specificity: *p* < 0.0001, χ^2^ = 29.85, *I*^2^ = 86.6%, Supplementary Figures S11A,B). The pooled DOR was 11.81 (95%CI 7.44–18.73), with no heterogeneity (*p* = 0.5561, Cochran-Q = 3.01, *I*^2^ = 0%, Supplementary Figure S11C). The AUC value was 0.84 ± 0.02 (Supplementary Figure S11D).

#### miRNA-125a

Four studies (Liu et al., 2020; Yang Y. et al., 2020; Zhao et al., 2020; Zhu, 2020) were included to analyze the overall predicting performance of miR-125a (Supplementary Figure S12). The pooled sensitivity was 0.86 (95%CI 0.79–0.91), with moderate heterogeneity (*p* = 0.0369, χ^2^ = 8.49, *I*^2^ = 64.7%, Supplementary Figure S12A). The pooled specificity was 0.48 (95%CI 0.43–0.53), with low heterogeneity (*p* = 0.2191, χ^2^ = 4.43, *I*^2^ = 32.2%, Supplementary Figure S12B). The pooled DOR was 5.69 (95%CI 2.50–12.95), with moderate heterogeneity (*p* = 0.0725, Cochran-Q = 6.98, *I*^2^ = 57.0%, Supplementary Figure S12C). The AUC value was 0.51 ± 0.12 (Supplementary Figure S12D).

#### miRNA-125b

Four studies (Xu et al., 2020; Zhao et al., 2020; Zhu, 2020; Zhang et al., 2024) assessed the predictive performance of microRNA-125b (Supplementary Figure S13). The pooled sensitivity was 0.68 (95%CI 0.60–0.76), with moderate heterogeneity (*p* = 0.0828, χ^2^ = 6.68, *I*^2^ = 55.1%, Supplementary Figure S13A). The pooled specificity was 0.76 (95%CI 0.71–0.80), with low heterogeneity (*p* = 0.2584, χ^2^ = 4.03, *I*^2^ = 25.5%, Supplementary Figure S13B). The pooled DOR was 6.36 (95%CI 4.18–9.68), with no heterogeneity (*p* = 0.2667, Cochran-Q = 3.95, *I*^2^ = 24.1%, Supplementary Figure S13C). The AUC value was 0.80 ± 0.03 (Supplementary Figure S13D).

#### miRNA-150

Four studies (Yang et al., 2022; Yang et al., 2015; Pan et al., 2021; Zhang et al., 2024) were included in order to analyze the overall predictive performance of microRNA-150 (see Supplementary Figure S14). The pooled sensitivity was 0.71 (95% CI 0.65–0.77), and the specificity was 0.70 (95% CI 0.66–0.74). Notably, significant heterogeneity was observed in the sensitivity analysis (sensitivity: *p* = 0.0024, χ^2^ = 14.37, *I*^2^ = 79.0%; specificity: *p* < 0.0001, χ^2^ = 25.07, *I*^2^ = 88.0%, Supplementary Figures S14A,B). The pooled DOR was 5.75 (95%CI 4.21–7.87), with no heterogeneity (*p* = 0.5495, Cochran-Q = 2.11, *I*^2^ = 0%, Supplementary Figure S14C). The AUC value was 0.76 ± 0.02 (Supplementary Figure S14D).

#### Sensitivity analysis and publication bias

**Figure 4:**
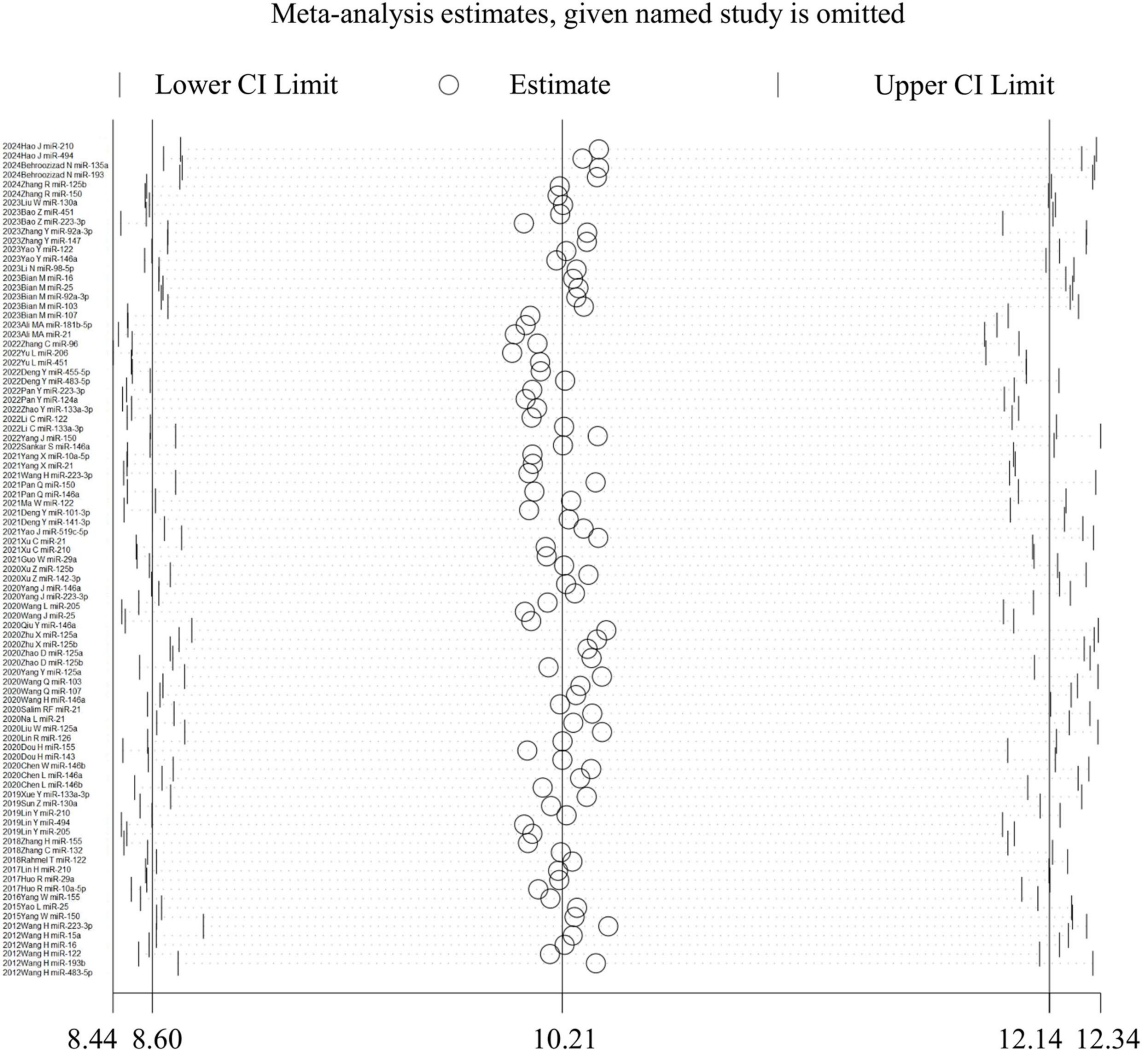
Sensitivity analysis for the results TmiRs. DOR, predicting odds ratio; ESS, effective sample size; miR, mircoRNA; TmiRs, total mixed miRNAs.

**Figure 5:**
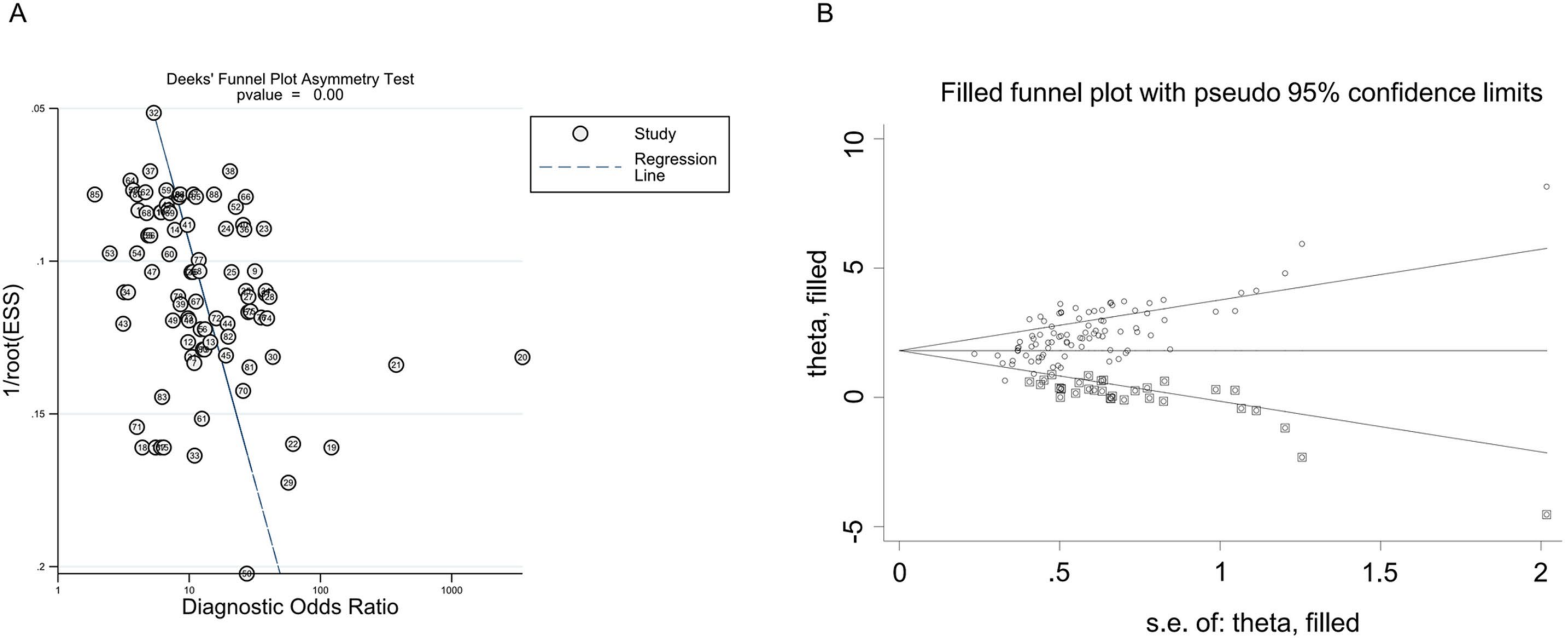
Publication bias of the individual trials on the results TmiRs. **(A)** Egger’s publication bias plots for the assessment of potential publication bias. Asymmetry of the dot distribution between regression lines showed potential publication bias, *p* < 0.001, *t* = 3.88, 95% CI 7.18–22.25. **(B)** The funnel plot of publication bias by the trim-and-fill method. After filled 28 potentially missing studies, the funnel plots were symmetrical. CI, confidence interval; DOR, predicting odds ratio; ESS, effective sample size; miR, mircoRNA; TmiRs, total mixed miRNAs.

None of the individual studies significantly affected the overall results, as confirmed by STATA 15.1 (Figure 4). The Egger’s regression test yielded a *p*-value of less than 0.001, a *t*-value of 3.88, and a 95% confidence interval ranging from 7.18 to 22.25 (Figure 5A). This finding suggests the presence of publication bias. However, the implementation of the trim-and-fill method to address publication bias revealed no substantial alteration in the findings when compared to the results obtained after the inclusion of 28 studies (*p* < 0.001, *Z* = 20.08, 95%CI 1.74–2.12), suggesting that the meta-analysis outcomes remained resilient to potential bias (Figure 5B).

## Discussion

Since their discovery approximately 10 years ago, numerous studies have investigated the potential of miRNAs as biomarkers for predicting sepsis mortality. In this meta-analysis, we have included 55 studies involving 2,047 non-survival and 4,396 survival sepsis patients, covering 41 different miRNAs. Our analysis revealed that TmiRs had a combined AUC of 0.83, with 76% sensitivity and 72% specificity, indicating that miRNAs exhibit moderate predictive accuracy as biomarkers for sepsis mortality. Furthermore, we conducted an assessment of individual miRNAs and identified that miR-133a-3p, miR-146a, miR-21, miR-210, miR-223-3p, miR-155, miR-25, miR-122, miR-125a, miR-125b, and miR-150 have been the most frequently examined in recent studies. Among these, miR-133a-3p exhibited the highest AUC in the SROC analysis, with a pooled sensitivity of 0.83 (95%CI 0.70–0.92), a pooled specificity of 0.79 (95%CI 0.71–0.86), and an SROC value of 0.90. Other microRNAs, including miR-146a, miR-21, miR-210, miR-223-3p, miR-155, miR-25, miR-122, miR-125a, miR-125b, and miR-150, also exhibited SROC values of 0.84, 0.87, 0.83, 0.89, 0.89, 0.89, 0.84, 0.51, 0.80, and 0.76, respectively. The findings of this study suggest that microRNAs (miRNAs), particularly miR-133a-3p, miR-146a, miR-21, miR-210, miR-223-3p, miR-155, miR-25, miR-122, miR-125b, and miR-150, have the potential to serve as useful biomarkers for predicting sepsis mortality. This meta-analysis is, to the best of our knowledge, the first to evaluate the accuracy of miRNAs in predicting sepsis mortality.

Considering the substantial heterogeneity observed in the analysis, a meta-regression was conducted to explore potential sources of variability. The analysis encompassed a comprehensive set of factors, including specimen type (serum, plasma, PBMCs), patient characteristics (neonates, children, adults), measurement techniques for microRNA (e.g., variations in qRT-PCR reference genes and extraction methods), geographical region, and total sample size. The analysis revealed that population differences and sample size contributed significantly to the heterogeneity, likely due to variations in immune responses and disease progression across different age groups and populations. Inconsistent sepsis diagnostic criteria (e.g., Sepsis 1.0, 2.0, 3.0) and methodological differences in RNA extraction and quantification further compounded the findings. Despite conducting subgroup analyses based on population and sample type, heterogeneity persisted, potentially due to the uneven distribution of studies. To address this challenge, a random-effects model was employed to mitigate the impact of heterogeneity. These findings underscore the necessity for standardized study designs in future research endeavors. The harmonization of diagnostic criteria, the standardization of measurement techniques, and the assurance of adequate sample sizes are imperative to mitigate bias and enhance the reliability of miRNAs as prognostic biomarkers in sepsis.

In a recent meta-analysis of 26 studies, Wang C. et al. (2022) evaluated the prognostic accuracy of the qSOFA, NEWS, and SIRS criteria for sepsis. Their findings indicated that qSOFA exhibited superior overall prognostic accuracy compared to SIRS and NEWS. The pooled sensitivity of qSOFA was 0.46 (95%CI 0.39–0.53), specificity was 0.82 (95%CI 0.76–0.86), and the AUC value was 0.69, which is lower than the results obtained in this study. Furthermore, several studies (Yang et al., 2022; Sun et al., 2019; Qiu et al., 2020; Wang L. et al., 2020; Ma et al., 2021; Wang H. et al., 2021; Xu et al., 2021) incorporated within the present meta-analysis assessed the collective predictive capacity of miRNAs, IL-6, PCT, CRP, SOFA score, and additional markers for sepsis mortality. Despite the unfeasibility of a comprehensive combined analysis of microRNAs (miRNAs) and other indicators, the integrated predictive approach employed in this study demonstrates considerable promise. Concurrent with this, previous studies (Yang et al., 2022; Wang H. et al., 2021; Xu et al., 2021; Li et al., 2022) underscored the significance of combined prediction in sepsis management, though further clinical research is necessary to elucidate its benefits.

Recent studies have underscored the critical roles of miRNAs and other non-coding RNAs (ncRNAs) in regulating immune responses and driving the progression of sepsis. MiRNAs such as miR-150, miR-146a, and miR-223 have been consistently associated with sepsis pathophysiology, with several studies confirming their potential as biomarkers for sepsis mortality (Formosa et al., 2022; Antonakos et al., 2022). Concurrently, the potential of miR-155, miR-21, miR-223-3p, miR-146a, and miR-125a as sepsis indicators has been previously demonstrated in our study (Zheng et al., 2023). The present study further corroborates the predictive value of miR-155, miR-21, miR-223-3p, and miR-146a, in addition to miR-125a, suggesting that these microRNAs may play pivotal roles in sepsis pathogenesis. Among these, miR-146a has been the subject of particular study, as evidenced by seven publications in our meta-analysis that affirm its predictive value for sepsis mortality. However, further research is needed to clarify the molecular mechanisms underlying its function. Future studies should prioritize exploring these microRNAs at the molecular level to enhance our understanding of their roles in sepsis. MiR-146a has been demonstrated to modulate immune responses during sepsis by targeting pivotal components of the NF-κB and TNF signaling pathways. It has been demonstrated that MiR-146a suppresses excessive inflammation by inhibiting the expression of interleukin-1 receptor-associated kinase (IRAK1) and TNF receptor-associated factor 6 (TRAF6), both of which are critical mediators in these pathways (Gilyazova et al., 2023). This regulatory function helps prevent an overactive immune response, which could otherwise lead to tissue damage and organ failure. Furthermore, it has been demonstrated that polymorphisms in miR-146a are associated with altered inflammatory responses, thereby reinforcing the complex interplay between genetic and environmental factors in the pathogenesis of these conditions.

In a similar manner, microRNA-133a-3p has been shown to offer protection against sepsis-induced acute respiratory distress syndrome (ARDS) by modulating SIRT1, a pivotal regulator of inflammation and oxidative stress. The downregulation of miR-133a-3p in septic patients has been associated with increased lung injury, while its upregulation has been linked to reduced inflammatory damage and improved lung function (Hui et al., 2024). These findings underscore the pivotal role of miR-133a-3p in moderating the inflammatory cascade in sepsis, thereby substantiating its potential as a therapeutic target.

MiR-150 has also been shown to modulate immune responses in septic patients (Formosa et al., 2022). Notably, the function of miR-150 in cancer is dual, serving as either a tumor suppressor or an oncogene, depending on the specific cancer type and the cellular context. For instance, in liver, ovarian, and colorectal cancers, miR-150 acts as a tumor suppressor, whereas in breast cancer it promotes tumor progression by regulating processes like epithelial-mesenchymal transition through matrix metalloproteinases and cell adhesion molecules (Ameri et al., 2023; Forterre et al., 2020). This dual role underscores the complexity of miR-150’s function across different diseases, including sepsis, where its dysregulation may significantly impact immune and inflammatory pathways (Mazziotta et al., 2023). Other microRNAs identified in our analysis, such as miR-125b and miR-193, have also been implicated in cancer and various other diseases, thereby reinforcing their broader regulatory roles (Forterre et al., 2020; Mazziotta et al., 2023). A comprehensive understanding of the roles of these miRNAs in both cancer and sepsis could offer valuable insights into their potential as biomarkers and therapeutic targets.

Furthermore, miR-223-3p has been shown to play a pivotal role in modulating immune responses during sepsis through multiple mechanisms. Specifically, it has been observed that miR-223-3p exerts its regulatory influence over autophagy in CD4+ T lymphocytes, a process that is achieved by directly targeting Forkhead box O1 (FOXO1). Overexpression of miR-223-3p has been shown to suppress FOXO1 expression, thereby reducing autophagic activity and preventing immune cell dysfunction in sepsis (Xiang et al., 2024). Moreover, the influence of miR-223-3p on the polarization of macrophages is characterized by its promotion of an anti-inflammatory M2 phenotype, thereby contributing to the alleviation of sepsis severity in experimental models (Dang and Leelahavanichkul, 2020). Another critical role of miR-223-3p is its suppression of the NLRP3 inflammasome, a key mediator of inflammatory responses, which reduces pro-inflammatory cytokine release and attenuates hyperinflammation (Shi et al., 2023). These findings underscore the central role of miR-223-3p in orchestrating both innate and adaptive immune responses during sepsis.

MiR-210 has been identified as a pivotal regulator of sepsis pathophysiology, particularly through its involvement in hypoxia-related pathways and immune responses. As a hypoxia-inducible microRNA, miR-210 is significantly upregulated in septic conditions, reflecting the tissue hypoxia characteristic of the disease. Elevated circulating levels of miR-210 have been demonstrated to be strongly associated with disease severity and mortality, underscoring its potential as a prognostic biomarker (Powell et al., 2022). Mechanistically, the disruption of mitochondrial function by miR-210 is attributed to its targeting of ISCU, a scaffold protein that is essential for iron–sulfur cluster assembly. This disruption leads to mitochondrial dysfunction, oxidative stress, and cardiomyocyte apoptosis, exacerbating myocardial injury (Chen et al., 2021). Furthermore, miR-210 has been shown to induce glycolytic reprogramming in macrophages, thereby enhancing pro-inflammatory cytokine production. This metabolic shift, while supporting acute inflammation, also contributes to systemic damage and multi-organ dysfunction (Virga et al., 2021). Collectively, these findings underscore the dual role of miR-210 in mediating inflammation and organ damage in sepsis.

Additionally, miR-122 plays a central role in sepsis by modulating immune responses, inflammation, and coagulation pathways, as well as protecting against organ damage. A recent study demonstrated that miR-122 mitigates sepsis-induced liver injury by targeting the BCL2A1 signaling pathway, thereby reducing macrophage apoptosis and alleviating inflammatory responses (Liu et al., 2024). Furthermore, the role of miR-122 extends to the regulation of pyroptosis, a form of programmed cell death associated with inflammation. By targeting NLRP1, miR-122-3p suppresses LPS-induced pyroptosis in macrophages, limiting the release of pro-inflammatory cytokines and reducing systemic inflammation (Li M. et al., 2023). Additionally, miR-122 influences coagulation and inflammatory pathways during sepsis. Through its interactions with MASP1 and HO-1 genes, miR-122-5p modulates coagulation abnormalities and systemic inflammation, demonstrating its dual role in regulating immune and hemostatic responses (Wang H. et al., 2022). Collectively, these findings emphasize the multifaceted functions of miR-122 in mitigating organ damage, controlling systemic inflammation, and regulating coagulation in sepsis, thereby establishing its potential as both a biomarker and a therapeutic target.

In addition to microRNAs (miRNAs), non-coding RNAs (ncRNAs) such as long non-coding RNAs (lncRNAs) have been found to regulate sepsis-induced organ dysfunction. Among these, MALAT1 and NEAT1 have garnered significant attention due to their distinct roles in modulating inflammatory pathways (Wang C. et al., 2021; Li et al., 2021). MALAT1 is associated with promoting inflammatory responses by facilitating NF-κB nuclear translocation, exacerbating lung injury during sepsis (Cui et al., 2021). Conversely, NEAT1 exhibits dual regulatory effects, either promoting or suppressing inflammation depending on its molecular interactions. For instance, NEAT1 acts as a molecular sponge for miR-124-3p, inhibiting STAT3-mediated pro-inflammatory signaling (Ghafouri-Fard et al., 2021). These findings highlight the intricate regulatory mechanisms of lncRNAs in sepsis, offering potential therapeutic targets warranting further investigation.

To enhance diagnostic accuracy, recent studies have investigated the potential of integrating miRNAs with traditional biomarkers such as IL-6, PCT, and CRP. The integration of multi-biomarker panels, encompassing miRNAs, has been shown to yield substantial improvements in diagnostic precision (Teggert et al., 2020). The integration of these multi-biomarker panels with miRNAs has been shown to yield substantial improvements in diagnostic accuracy (Teggert et al., 2020; Bradley and Bhalla, 2023). Future research should prioritize validating these multi-biomarker panels in clinical settings to maximize their utility.

In our meta-analysis, we comprehensively examined studies that utilized diverse sample types, including plasma, PBMCs, and whole blood, as outlined in Table 1. This diversity enables a comprehensive evaluation of the predictive utility of miRNAs across various biological sources. However, due to the limited number of studies focusing on PBMCs, the findings predominantly reflect circulating miRNAs found in plasma and serum. Circulating miRNAs are primarily transported in extracellular vesicles (EVs), where they are shielded from degradation and can function as systemic signaling molecules, mirroring systemic inflammatory and immune responses in sepsis (Real et al., 2018). For instance, studies have demonstrated that plasma-derived EVs in septic patients carry miRNAs implicated in inflammation and cell cycle regulation, underscoring their significance in sepsis pathophysiology (Real et al., 2018). Conversely, PBMC-derived miRNAs provide insights into cell-specific regulatory processes, such as immune cell activation. While underrepresented in this study, PBMC-derived miRNAs warrant further exploration to uncover their potential role in sepsis pathophysiology (Antonakos et al., 2022). Integrating data from both circulating and PBMC-derived miRNAs could advance diagnostic and therapeutic approaches (Xu et al., 2018).

The present meta-analysis has several notable strengths. Firstly, it is the inaugural meta-analysis to systematically evaluate the prognostic accuracy of miRNAs in predicting sepsis mortality. Secondly, it encompasses a substantial number of studies, with 55 studies included, which is more than most previous meta-analyses on sepsis mortality prediction. Thirdly, we evaluated the accuracy of nine miRNAs—namely, miR-133a-3p, miR-146a, miR-21, miR-210, miR-223-3p, miR-155, miR-25, miR-122, and miR-125b—that have the potential to serve as sepsis biomarkers.

While the present study underscores the potential of miRNAs as biomarkers for sepsis, several challenges exist that limit their clinical applicability. Variability in the expression of miRNAs across diverse populations, influenced by factors such as age, underlying health conditions, and disease progression, may compromise diagnostic accuracy and reduce generalizability. Furthermore, the evidence supporting each individual miRNA indicator is derived from a limited number of studies, ranging from three to seven, which introduces potential bias. The lack of standardized methodologies for detecting miRNAs, including inconsistencies in sample collection, RNA extraction, and qRT-PCR reference genes, adds another layer of complexity. Although meta-regression has been employed to address some of these issues, significant heterogeneity remains due to differences in the indicators of miRNAs and the study methodologies. Moreover, the predominance of studies conducted in Asian populations may limit the generalizability of our findings, considering the well-documented disparities in genetics and healthcare practices across different regions. To enhance the reproducibility and global relevance of these biomarkers, future studies should aim to incorporate more diverse populations and adopt standardized protocols.

## Conclusion

In summary, our meta-analysis demonstrated that microRNAs (miRNAs), particularly miR-133a-3p, miR-155-5p, miR-146a, miR-21, miR-210, miR-223-3p, and miR-155, could serve as useful biomarkers for predicting sepsis mortality. To improve reliability, future research should focus on standardizing protocols, conducting longitudinal studies, and developing subgroup-specific miRNA panels for neonates, children, and adults. These advancements are crucial for transforming miRNAs into robust and universally applicable biomarkers in sepsis management.

## Data Availability

The original contributions presented in the study are included in the article/Supplementary material, further inquiries can be directed to the corresponding author.

## Glossary

CRP: C-reactive protein
PCT: Procalcitonin
miRNAs: microRNAs
CI: Confidence interval
SROC: Summary receiver operating characteristic curves value
AUC: Area under the curve
qSOFA: Quick sequential organ failure assessment score
NEWS: National early warning score
PRISMA: Preferred reporting items for systematic reviews and meta-analyses
CNKI: China national knowledge infrastructure
CENTRAL: Cochrane central register of controlled trials
ROC: Receiver operating characteristic
TP: True positive false positive
FP: False positive
FN: False negative
TN: True negative
QUADAS: Quality assessment of diagnostic accuracy studies
DOR: Diagnostic odds ratio
I^2^: Inconsistency index
TSA: Trial sequential analysis
RIS: Required information size
PBMCs: Peripheral blood mononuclear cells
SIRS: Systemic inflammatory response syndrome
TmiRs: Total mixed miRNAs
OR: Odds ratio
ESS: Effective sample size
HC: Healthy control
N/R: Not report
SE: Standard error
