# Supplementary material for: Significances of miRNAs for predicting sepsis mortality: a meta-analysis
Source: Front Microbiol. 2025 Mar 11;16:1472124. doi: 10.3389/fmicb.2025.1472124 (PMC11933020; doi:10.3389/fmicb.2025.1472124)
Supplement: Supplementary file 1 [file Data_Sheet_1.pdf]

Supplementary Materials of "Significances of miRNAs for predicting sepsis mortality: a meta-analysis"

Supplementary Table 1. PRISMA checklist.

**Section/topic**	**#**	**Checklist item**	**Location in manuscript**
Title	1	Significances of miRNAs for predicting sepsis mortality: a meta-analysis	Title
ABSTRACT			
Structured summary	2	Background: Sepsis is a life-threatening condition caused by a dysregulated immune response to infection and remains a major cause of mortality in intensive care units (ICUs). Recent studies have identified microRNAs (miRNAs), a class of small RNA molecules, as potential biomarkers for diagnosing and predicting outcomes in sepsis patients. However, the results of these studies have been inconsistent. This meta-analysis aims to comprehensively evaluate the diagnostic and prognostic value of miRNAs in predicting sepsis-related mortality.<br>Methods: A comprehensive literature search was performed across major databases, including PubMed, Cochrane Library, EMBASE, and CNKI, up to April 7, 2024. Data extraction and meta-analysis were conducted using Meta-disc 1.4 and STATA 15.1, employing both fixed- and random-effects models to ensure robust statistical analysis.<br>Results: A total of 55 studies met the inclusion criteria and were analyzed. The pooled sensitivity, specificity, and area under the summary receiver operating characteristic (SROC) curve for miRNA detection were calculated. The overall performance of total miRNA detection demonstrated a sensitivity of 0.76 (95% confidence interval [CI]: 0.74-0.77), a specificity of 0.72 (95% CI: 0.71-0.73), and an SROC value of 0.83. Subgroup analyses revealed that miR-133a-3p exhibited the highest diagnostic accuracy, with a pooled sensitivity of 0.83 (95% CI: 0.70-0.92), specificity of 0.79 (95% CI: 0.71-0.86), and an SROC value of 0.90. Additionally, other miRNAs, including miR-146a, miR-21, miR-210, miR-223-3p, miR-155, miR-25, miR-122, miR-125a, miR-125b, and miR-150, also demonstrated high SROC values (0.84 to 0.76).<br>Conclusions: This meta-analysis underscores the potential	Abstract

		of several microRNAs (miRNAs) as reliable biomarkers for predicting sepsis mortality. Specifically, miR-133a-3p, miR-146a, miR-21, miR-210, miR-223-3p, miR-155, miR-25, miR-122, miR-125b, and miR-150 emerge as promising candidates for clinical applications in sepsis prognosis.	
INTRODUCTION			
Rationale	3	Sepsis is a leading cause of mortality in Intensive Care Units (ICUs). It is characterized by a dysregulated immune response to infection that often results in organ failure and high mortality. Early identification of patients at high risk for sepsis is therefore vital for reducing mortality and improving outcomes. Current diagnostic and management strategies rely on clinical assessments, monitoring of vital signs, and laboratory parameters, supported by scoring systems such as the Quick Sequential Organ Failure Assessment Score (qSOFA) and the National Early Warning Score (NEWS). Despite their utility, these systems have inherent limitations, necessitating continuous clinical monitoring and highlighting the urgent need for novel predictive indicators of sepsis mortality.<br>MicroRNAs (miRNAs), a class of small non-coding RNA molecules, have gained attention for their significant role in the regulation of gene expression and immune responses in sepsis. A recent meta-analysis validated the diagnostic utility of miRNAs and identified miR-223-3p as a potential biomarker for sepsis. However, the predictive value of miRNAs in sepsis mortality remains controversial due to inconsistent findings across studies.	Introduction
Objectives	4	We collected all published case-control studies to gather evidence on how the diagnostic performance of miRNAs distinguished sepsis mortality.	Introduction
METHODS			
Protocol and registration	5	This analysis was carried out according to a predetermined protocol, as recommended by Deeks. For data collection and analysis, the Preferred Reporting Items for Systematic Reviews and Meta-Analyses (PRISMA) Statement was utilized. This study was registered with PROSPERO (CRD42024530167).	Materials and Methods
Eligibility criteria	6	Criteria for inclusion: (1) all sepsis patients were confirmed by diagnosis criteria; (2) trials evaluating the expression of miRNAs; (3) contained data of receiver operating characteristic (ROC) curve and the essential sample size; (4) all reports had specific numbers of sepsis patients who	Materials and Methods

		died and survived; (5) full text published in English or Chinese.<br>Criteria for exclusion: (1) reviews, letters, conferences articles, or case reports; (2) no adequate data to analysis; (3) duplicated studies.	
Information sources	7	To find relevant studies, we searched PubMed, EMBASE, China National Knowledge Infrastructure (CNKI), and the Cochrane Central Register of Controlled Trials databases until April 7, 2024.	Materials and Methods
Search	8	Search terms were ('sepsis' OR 'septicemia' OR 'pyemia') AND ('MicroRNAs' OR 'MicroRNA' OR 'miRNAs' OR 'miRNA'). PubMed database was searched as follows: (Sepsis[MeSH Terms] OR septicemia OR pyemia) AND (MicroRNAs[MeSH Terms] OR MicroRNA OR miRNAs OR miRNA). Search terms for EMBASE, Cochrane and CNKI with corresponding publication numbers can be found in the S Appendix. Language was limited in English and Chinese.	Materials and Methods
Study selection	9	First, we checked at the study titles and abstracts. The full articles of possibly relevant studies were then retrieved and verified for compliance with the inclusion and exclusion criteria.	Materials and Methods
Data collection process	10	Two investigators (Mou Peng and Yue Zhang) used the inclusion and exclusion criteria independently to review report eligibility at the title and abstract level, with third reviewer (Yifei Li) determining the divergences and study quality. Two investigators (Yue Zhang and Xiaolan Zheng) independently assessed all enrolled reports according to the Quality Assessment of Diagnostic Accuracy Studies 2 (QUADAS-2) list(13), and any disagreements were resolved through discussion with a third reviewer (Yifei Li). Finally, two researchers (Xiaolan Zheng and Mou Peng) retrieved data that may be used to determine true positive (TP), false positive (FP), false negative (FN), and true negative (TN), including sensitivity, specificity, and the number of patients.	Materials and Methods
Data items	11	the data of true positive, false positive, false negative, and true negative; or the data of the receiver operating characteristic (ROC) curve, and essential sample size	Materials and Methods
Risk of bias in individual studies	12	We used Stata statistical software (STATA, version 15.1) to obtain a quantitative analysis of all the publication bias according to funnel plots and the Deek's test. An asymmetric distribution of data points in the funnel plot	Materials and Methods

		with a quantified result of P<.05 indicated the presence of potential publication bias	
Summary measures	13	The following indicators of different types of miRNAs were measured: sensitivity, specificity, diagnostic odds ratio (DOR), and area under the summary receiver operating characteristic curves value (SROC).	Materials and Methods
Synthesis of results	14	Sensitivity, specificity, diagnostic odds ratio (DOR), and area under the summary receiver operating characteristic curves value (SROC).	Materials and Methods
Risk of bias across studies	15	A quantitative study of all the publication bias was undertaken using STATA version 15.1 after funnel plots and the Deeks' test (Stata Corporation, College Station, Texas, USA). An uneven distribution of data points with a quantified result of P 0.05 raised the probability of publishing bias. We apply the trim-and-fill method to assess the influence of publication bias on the outcomes when publication bias occurs. Even if there is publication bias, if there is no significant difference between the results before and after the trim-and-fill method test, the results are stable and dependable.	Materials and Methods
Additional analyses	16	We carried out the meta-regression analysis using STATA 15.1 to detect where the potential factor for heterogeneity origin from. Sensitivity analysis was conducted for every study to determine the influence of individual trials on the results, using STATA 15.1 for meta-analysis fixed/random-effects estimates. Meta-Disc 1.4 was used to detect threshold effects in studies and conduct subgroup analysis.	Materials and Methods
RESULTS			
Study selection	17	Initially, the search method retrieved 3912 potentially relevant papers, of which 249 articles were considered to read their whole articles after assessing titles and abstracts. However, due to article types, 22 papers were removed, and 119 papers lacked data on TP, FN, FP, and TN. Furthermore, 53 articles did not include a comparison of survival and non-survival sepsis patients. Fig 1 presents the study selection procedure.	Results
Study characteristics	18	Finally, the meta-analysis comprised 55 studies(3, 6, 7, 19-70), totaling 6443 sepsis patients, including 2047 non-survival patients and 4396 survival patients, and involving 41 miRNAs. 11 miRNAs (miR-133a-3p, miR-146a, miR-21, miR-210, miR-223-3p, miR-155, miR-25, miR-122, miR-125a, miR-125b, and miR-150) were found to be implicated in more than two investigations. Furthermore, the age of the population was diverse. Four studies focused	Results

		on newborns less than 28 days old, five on children older than 1 month, and the remaining 46 on adults. Additionally, the sample types of 26 studies were plasma, 25 studies were serum, two reports were peripheral blood mononuclear cells (PBMC), and two studies were whole blood. Moreover, 52 reports from Asian (50 from China, one from India, and one from Iran), two from Africa (Egypt), and one from Europe (Germany). Furthermore, the 30 studies had a larger overall sample size ($n \geq 100$) than the remaining 25 ($n < 100$). Among the included articles, two reports followed the criteria for sepsis diagnosis were derived from sepsis 1.0(71), 13 from sepsis 2.0(72), and 38 from sepsis 3.0(2), while the remaining two articles did not give precise predicting criteria versions. Besides, 41 articles used U6 as a qRT-PCR reference gene, eight studies used non-U6, and six studies did not specify which reference gene was used. Table 1 shows the essential characteristics of the articles that were included.	
Risk of bias within studies	19	None of the investigations had a significant impact, and STATA 15.1 confirmed the TmiRs findings (Fig 4). Furthermore, the asymmetric distribution of the Egger's regression test result suggested that there was publication bias ($P < 0.001$, $t = 3.88$, 95% CI 7.18-22.25, Fig 5A). The trim-and-fill method is then used to assess the effect of publication bias on the results, and revealed no significant difference between the results before ($P < 0.001$, $Z = 26.42$, 95% CI 2.15-2.50) and after filled 28 studies ($P < 0.001$, $Z = 20.08$, 95% CI 1.74-2.12) (Fig 5B), implying that the meta-analysis results were robust even with publication bias.	Results
Results of individual studies	20	We discovered that TmiRs had a combined AUC of 0.83, with 76% sensitivity and 72% specificity, indicating that miRNAs had a moderate predicting accuracy as a predicting biomarker in sepsis mortality. Besides, we examined individual miRNAs in the overall miRNA library and discovered that miR-133a-3p, miR-146a, miR-21, miR-210, miR-223-3p, miR-155, miR-25, miR-122, miR-125a, miR-125b, and miR-150 were the most often used in recent studies. Among them, miR-133a-3p group had the highest AUC of SROC among all miRNAs: pooled sensitivity, 0.83(95%CI 0.70-0.92); pooled specificity, 0.79 (95%CI 0.71-0.86); and SROC, 0.90. MiR-146a, miR-21, miR-210, miR-223-3p, miR-155, miR-25, miR-122, miR-125a, miR-125b, and miR-150 had SROC values of 0.84,	Results

		0.87, 0.83, 0.89, 0.89, 0.89, 0.84, 0.51, 0.80 and 0.76, respectively.	
Synthesis of results	21	The overall predicting assessment of total mixed miRNA (TmiR) in identifying sepsis has been summarized in Fig 2. The summary sensitivity was 0.76 (95%CI 0.74-0.77), with a significant heterogeneity (P < 0.0001, x2 = 357.42, I2 = 75.4%, Fig 2A). The pooled specificity was 0.72 (95%CI 0.71-0.73), and the pooled estimation also showed noticeable heterogeneity (P < 0.0001, x2 = 901.34, I2 = 90.2%, Fig 2B). In addition, the pooled DOR was 10.21 (95% CI 8.60-12.14) with a significant heterogeneity (P < 0.0001, Cochran-Q = 218.42, I2 = 59.7%, Fig 2C). The calculated AUC value was 0.83 (Fig 2D). Besides, we examined individual miRNAs in the overall miRNA library and discovered that miR-133a-3p, miR-146a, miR-21, miR-210, miR-223-3p, miR-155, miR-25, miR-122, miR-125a, miR-125b, and miR-150 were the most often used in recent studies. Among them, miR-133a-3p group had the highest AUC of SROC among all miRNAs: pooled sensitivity, 0.83(95%CI 0.70-0.92); pooled specificity, 0.79 (95%CI 0.71-0.86); and SROC, 0.90. MiR-146a, miR-21, miR-210, miR-223-3p, miR-155, miR-25, miR-122, miR-125a, miR-125b, and miR-150 had SROC values of 0.84, 0.87, 0.83, 0.89, 0.89, 0.89, 0.84, 0.51, 0.80 and 0.76, respectively.	Results
Risk of bias across studies	22	None of the investigations had a significant impact, and STATA 15.1 confirmed the TmiRs findings (Fig 4). Furthermore, the asymmetric distribution of the Egger's regression test result suggested that there was publication bias (P < 0.001, t = 3.88, 95% CI 7.18-22.25, Fig 5A). The trim-and-fill method is then used to assess the effect of publication bias on the results, and revealed no significant difference between the results before (P < 0.001, Z = 26.42, 95% CI 2.15-2.50) and after filled 28 studies (P < 0.001, Z = 20.08, 95% CI 1.74-2.12) (Fig 5B), implying that the meta-analysis results were robust even with publication bias.	Results
Additional analysis	23	We ran a meta-regression analysis to see what factors might be causing the heterogeneities. Type of samples, region, sepsis diagnostic criteria, qRT-PCR reference genes, patient age structure, follow-up time, miRNA expression level (up or down), and the total sample size were all evaluate in the meta-regression. According to the findings (Fig 3), the population (P = 0.013, t = -2.53, 95%CI 0.39-	Results

		0.89, Fig 3A) and the total sample size (P = 0.003, t = -3.01, 95%CI 0.40-0.83, Fig 3B) may be the source of heterogeneity, While the remaining six factors are not (P > 0.05, Fig 3C-H).	
**DISCUSSION**			
Summary of evidence	24	In this meta-analysis, we enrolled 55 studies totaling 2047 non-survival and 4396 survival sepsis patients, involving 41 miRNAs. Finally, we discovered that TmiRs had a combined AUC of 0.83, with 76% sensitivity and 72% specificity, indicating that miRNAs had a moderate predicting accuracy as a predicting biomarker in sepsis mortality. Besides, we examined individual miRNAs in the overall miRNA library and discovered that miR-133a-3p, miR-146a, miR-21, miR-210, miR-223-3p, miR-155, miR-25, miR-122, miR-125a, miR-125b, and miR-150 were the most often used in recent studies. Among them, miR-133a-3p group had the highest AUC of SROC among all miRNAs: pooled sensitivity, 0.83(95%CI 0.70-0.92); pooled specificity, 0.79 (95%CI 0.71-0.86); and SROC, 0.90. MiR-146a, miR-21, miR-210, miR-223-3p, miR-155, miR-25, miR-122, miR-125a, miR-125b, and miR-150 had SROC values of 0.84, 0.87, 0.83, 0.89, 0.89, 0.89, 0.84, 0.51, 0.80 and 0.76, respectively. These results revealed that miRNAs, specifically miR-133a-3p, miR-146a, miR-21, miR-210, miR-223-3p, miR-155, miR-25, miR-122, miR-125b, and miR-150 could be consider as useful biomarkers for predicting sepsis mortality.	Discussion
Limitations	25	Variability in miRNA expression across different populations, influenced by factors such as age, underlying health conditions, and disease progression, may compromise diagnostic accuracy and reduce generalizability. Furthermore, each miRNA indicator was supported by only three to seven studies, which may introduce bias. The lack of standardized methodologies for miRNA detection, including inconsistencies in sample collection, RNA extraction, and qRT-PCR reference genes, adds another layer of complexity. Although meta-regression helped address some of these issues, significant heterogeneity remains due to differences in miRNA indicators and study methodologies. Additionally, most included studies were conducted in Asian populations, which may limit the external validity of our findings given regional differences in genetics and healthcare practices. Future studies should aim to incorporate more diverse	Discussion

		populations and adopt standardized protocols to enhance the reproducibility and global relevance of miRNA biomarkers in sepsis diagnostics.	
Conclusions	26	In summary, our meta-analysis demonstrated that microRNAs (miRNAs), particularly miR-133a-3p, miR-155-5p, miR-146a, miR-21, miR-210, miR-223-3p, and miR-155, could serve as useful biomarkers for predicting sepsis mortality. To improve reliability, future research should focus on standardizing protocols, conducting longitudinal studies, and developing subgroup-specific miRNA panels for neonates, children, and adults. These advancements are crucial for transforming miRNAs into robust and universally applicable biomarkers in sepsis management.	Conclusions
Funding	27	None.	

From: Moher D, Liberati A, Tetzlaff J, Altman DG, The PRISMA Group (2009). Preferred Reporting Items for Systematic Reviews and Meta-Analyses: The PRISMA Statement. PLoS Med 6(7): e1000097. doi:10.1371/journal.pmed1000097 For more information, visit: **www.prisma-statement.org**.

Supplementary Table 2. Summary of Sensitivity Analysis Results for Various Exclusion Conditions.

Group	Exclusion Condition	Combined OR	95% CI	I^2	p-value	Tau-squared
Region	Asian	64.299	8.251-501.083	77.7%	0.004	3.1200
	Africa	9.875	8.349-11.680	57.5%	0.000	0.3348
	Europe	10.208	8.577-12.148	60.1%	0.000	0.3816
Population	Neonates	9.779	8.261-11.576	57.8%	0.000	0.3333
	Children	9.977	8.299-11.993	61.1%	0.000	0.3969
	Adults	16.888	10.227-27.886	50.4%	0.019	0.3927
Diagnostic criteria	Sepsis1.0	10.318	8.650-12.308	59.4%	0.000	0.3677
	Sepsis2.0	10.193	8.348-12.447	57.1%	0.000	0.3427
	Sepsis3.0	10.936	7.815-15.305	69.5%	0.000	0.6093
	N/R	9.887	8.359-11.694	57.5%	0.000	0.3350
Follow-up time	< 1month	9.260	4.075-21.038	69.4%	0.006	0.7234
	1-3months	10.088	8.475-12.008	59.9%	0.000	0.3762
	N/R	10.418	8.744-12.413	59.2%	0.000	0.3667
qRT-PCR	U6	11.828	7.714-18.138	62.1%	0.000	0.5381

reference genes	Non-U6	10.006	8.377-11.951	58.8%	0.000	0.3424
	N/R	10.124	8.436-12.149	60.3%	0.000	0.3875
miRNA expression level	Up	7.490	6.044-9.281	25.2%	0.110	0.0801
	Down	11.812	9.371-14.889	66.1%	0.000	0.5161
Type of samples	Serum	8.201	6.665-10.091	49.7%	0.000	0.2445
	Plasma	12.723	9.980-16.219	59.5%	0.000	0.4254
	PBMCs	16.582	7.699-35.715	5.9%	0.346	0.0273
	Whole blood	9.065	5.015-16.385	18.9%	0.286	0.1201
Total sample size	< 100	8.419	6.896-10.279	62.5%	0.000	0.3156
	≥ 100	14.724	11.009-19.693	41.3%	0.005	0.3315

OR = odds ratio, CI = confidence interval, N/R = not report.

Supplementary Appendix: Search strategies for EMBASE (A), the Cochrane Central Register of Controlled Trials (B), and China National Knowledge Infrastructure (C).

A: Embase database was searched as follows: #1 'sepsis' OR 'pyemia' OR 'septicemia', #2 'MicroRNAs' OR 'MicroRNA' OR 'miRNAs' OR 'miRNA', #1 AND #2.

Embase		
1	('sepsis' or 'pyemia' or 'septicemia').mp. [mp=title, abstract, heading word, drug trade name, original title, device manufacturer, drug manufacturer, device trade name, keyword, floating subheading word, candidate term word]	395867
2	('MicroRNAs' or 'MicroRNA' or 'miRNAs' or 'miRNA').mp. [mp=title, abstract, heading word, drug trade name, original title, device manufacturer, drug manufacturer, device trade name, keyword, floating subheading word, candidate term word]	249298
3	#1 AND #2	1993

B: the Cochrane Central Register of Controlled Trials database was searched as follows: (‘sepsis’ OR ‘pyemia’ OR ‘septicemia’) AND (‘MicroRNAs’ OR ‘MicroRNA’ OR ‘miRNAs’ OR ‘miRNA’) in Title Abstract Keyword

the Cochrane Central Register of Controlled Trials		
1	(‘sepsis’ OR ‘pyemia’ OR ‘septicemia’) and (‘MicroRNAs’ OR ‘MicroRNA’ OR ‘miRNAs’ OR ‘miRNA’)in Title Abstract Keyword	14604
2	(‘MicroRNAs’ OR ‘MicroRNA’ OR ‘miRNAs’ OR ‘miRNA’) in Title Abstract Keyword	1591
3	#1 AND #2	11

C: China National Knowledge Infrastructure database was searched as follows: (sepsis OR pyemia OR septicemia) AND (MicroRNAs OR MicroRNA OR miRNAs OR miRNA) in Title Abstract Keyword.

China National Knowledge Infrastructure database		
1	(sepsis OR pyemia OR septicemia) in Title Abstract Keyword	246050
2	(MicroRNAs OR MicroRNA OR miRNAs OR miRNA) in Title Abstract Keyword	235857
3	#1 AND #2	1401

Supplementary Figures:

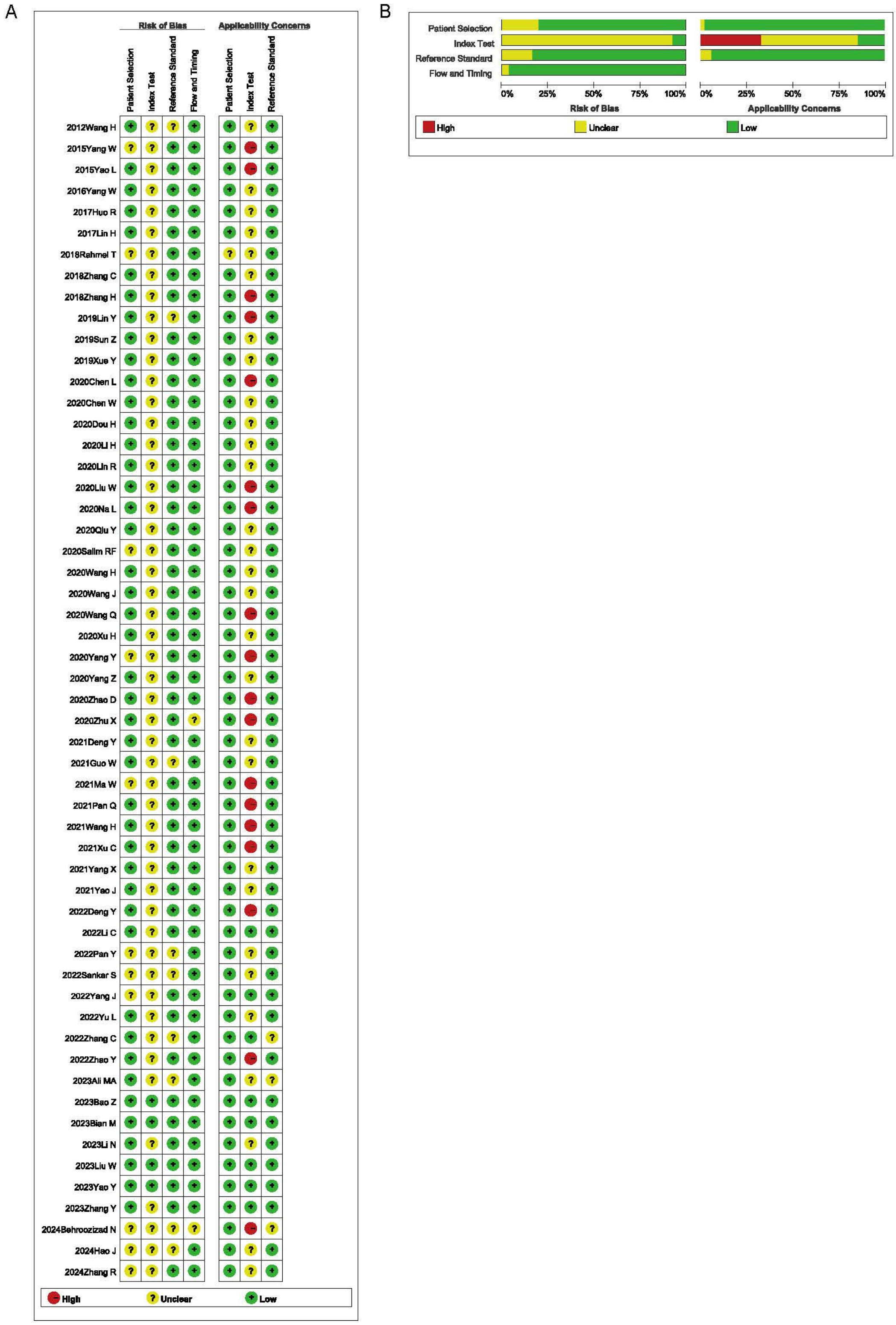

Supplementary Fig 1. Quality assessment of the included studies according to QUADAS-2. (A) Methodological quality graph; (B) Methodological quality summary.

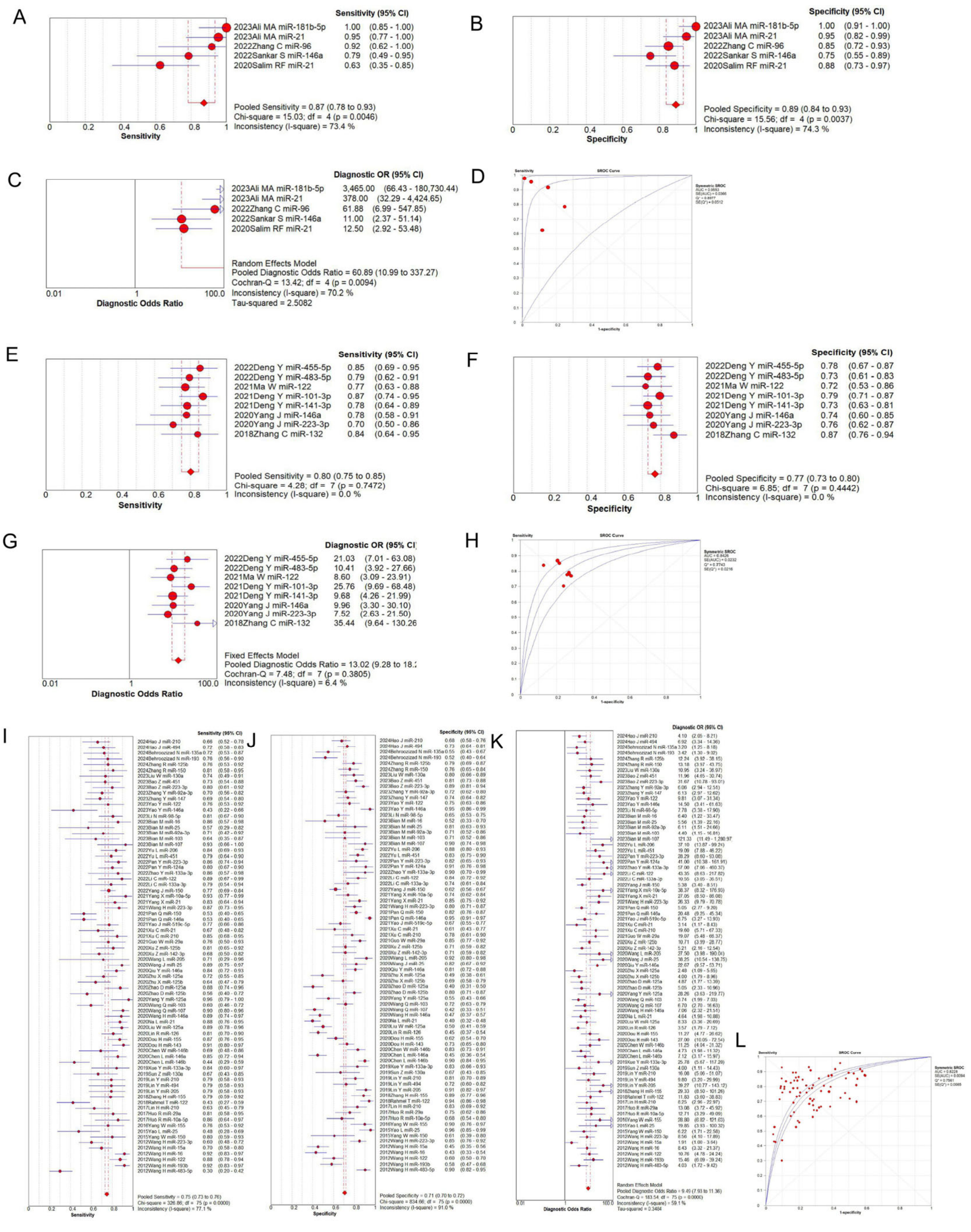

Supplementary Fig 2. Performance of population for sepsis diagnosis. (A) Pooled sensitivity of neonates. (B) Pooled specificity of neonates. (C) Total DOR of neonates. (D) The SROCs of neonates. (E) Pooled sensitivity of children (F) Pooled specificity of children. (G) Total DOR of children. (H) The SROCs of children. (I) Pooled sensitivity of adults. (J) Pooled specificity of adults. (K) Total DOR of adults. (L) The SROCs of adults. CI = confidence interval, DOR=predicting odds ratio, miR = mircoRNA, OR = odds ratio, SROC = summary receiver operating characteristic curves value.

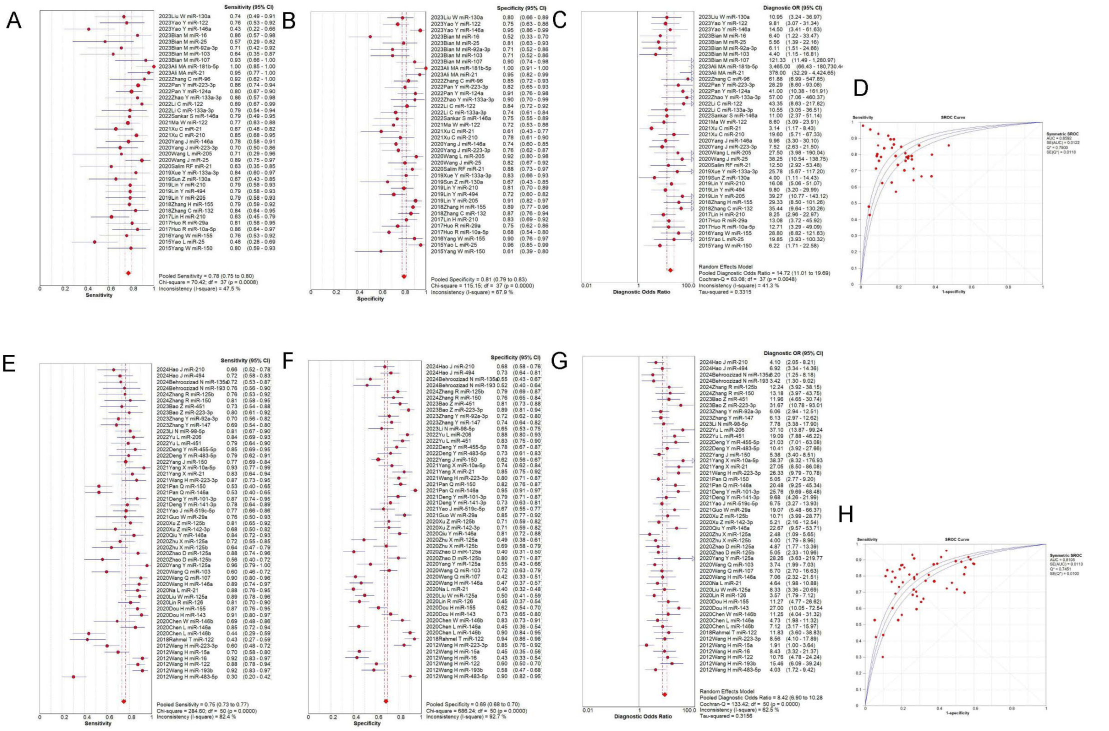

Supplementary Fig 3. Performance of sample size detection for sepsis diagnosis. (A) Pooled sensitivity of small sample size (n < 100). (B) Pooled specificity of small sample size (n < 100). (C) Total DOR of small sample size (n < 100). (D) The SROCs of small sample size (n < 100). (E) Pooled sensitivity of large sample size (n ≥ 100). (F) Pooled specificity of large sample size (n ≥ 100). (G) Total DOR of large sample size (n ≥ 100). (H) The SROCs of large sample size (n ≥ 100). CI = confidence interval, DOR=predicting odds ratio, miR = mircoRNA, OR = odds ratio, SROC = summary receiver operating characteristic curves value.

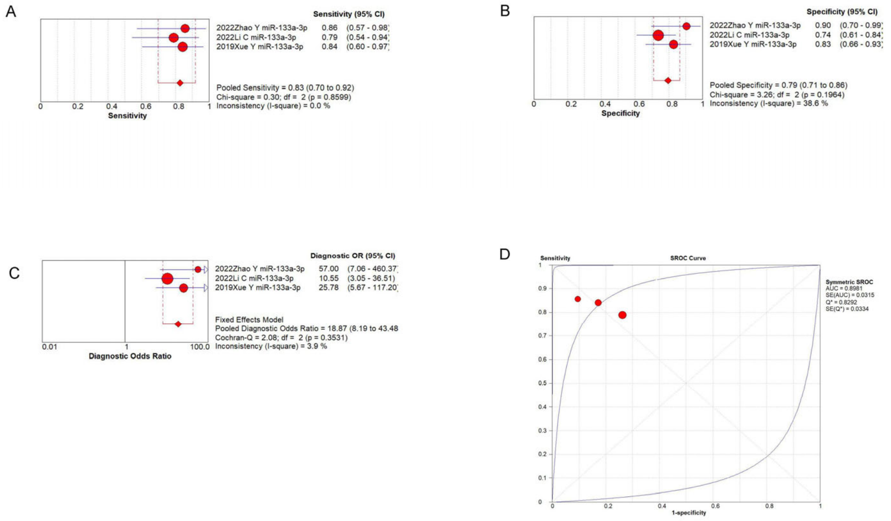

Supplementary Fig 4. Performance of miR-133a-3p detection for sepsis diagnosis. (A) Pooled sensitivity. (B) Pooled specificity. (C) Overall DOR. (D) The SROCs for all datasets.

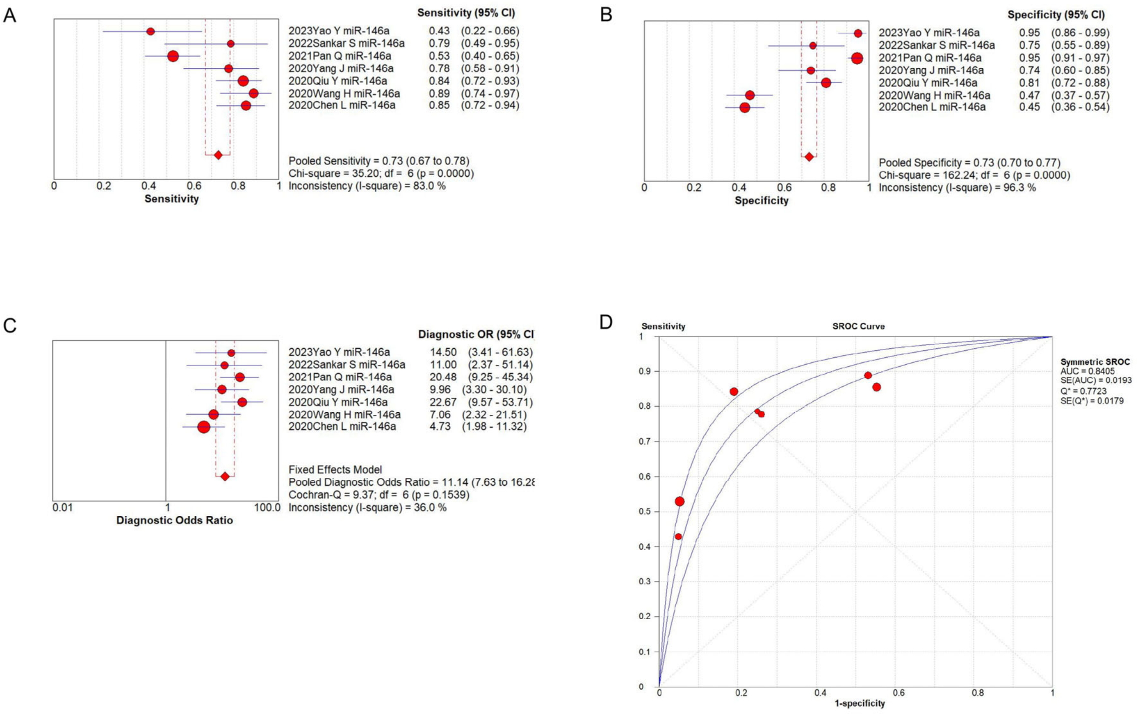

Supplementary Fig 5. Performance of miR-146a detection for sepsis diagnosis. (A) Pooled sensitivity. (B) Pooled specificity. (C) Overall DOR. (D) The SROCs for all datasets.

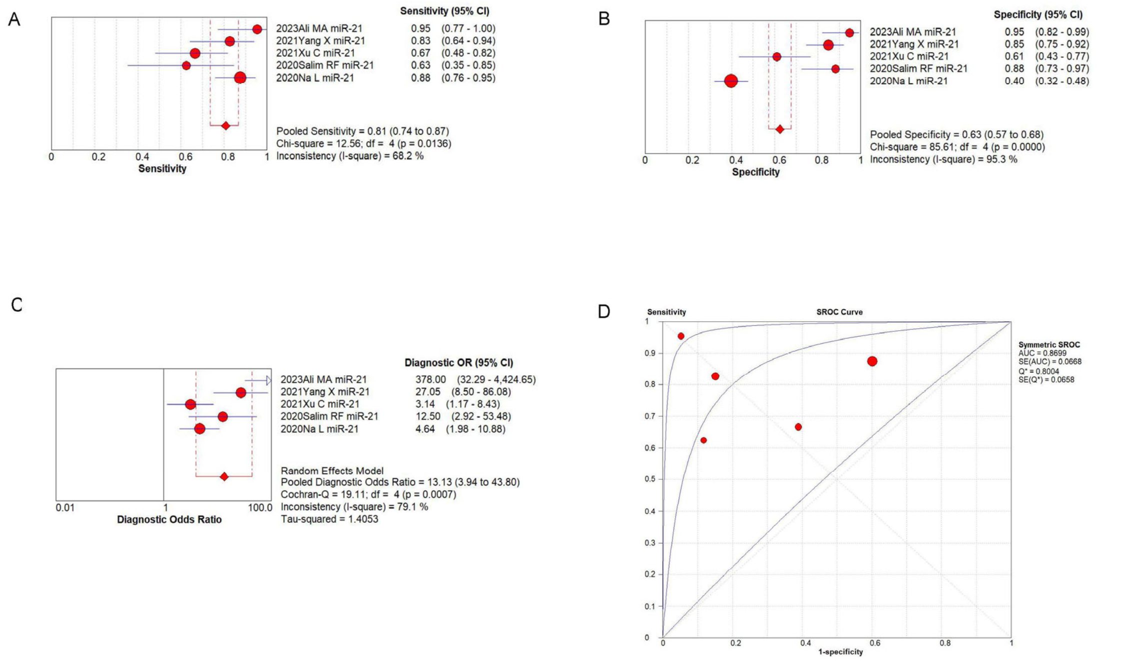

Supplementary Fig 6. Performance of miR-21 detection for sepsis diagnosis. (A) Pooled sensitivity. (B) Pooled specificity. (C) Overall DOR. (D) The SROCs for all datasets.

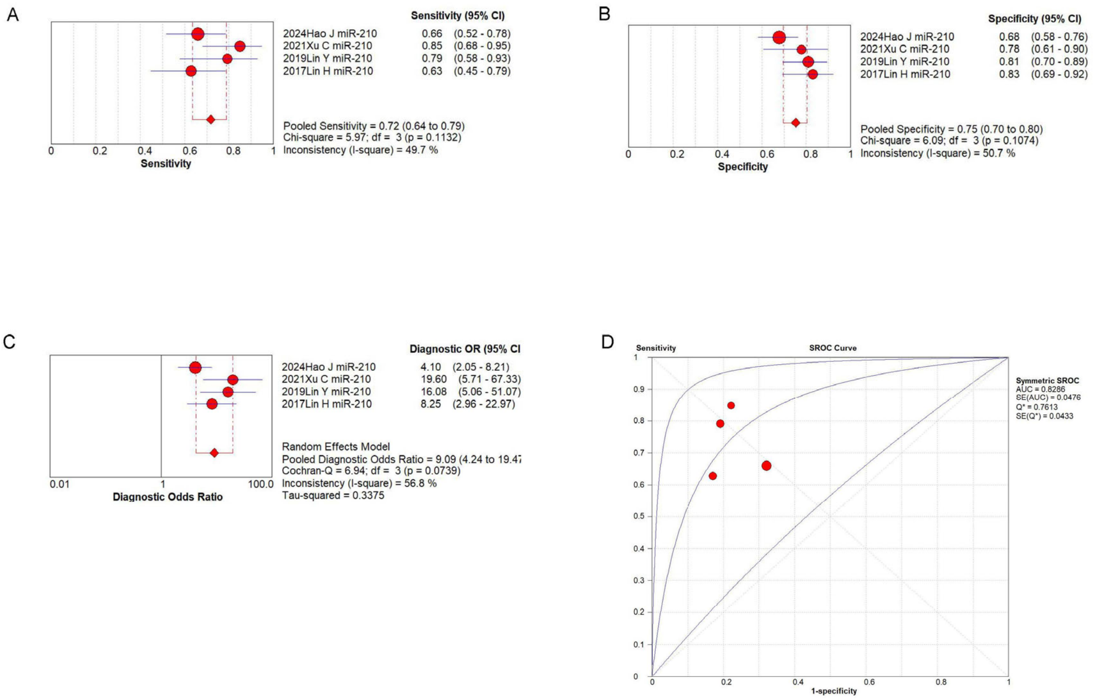

Supplementary Fig 7. Performance of miR-210 detection for sepsis diagnosis. (A) Pooled sensitivity. (B) Pooled specificity. (C) Overall DOR. (D) The SROCs for all datasets.

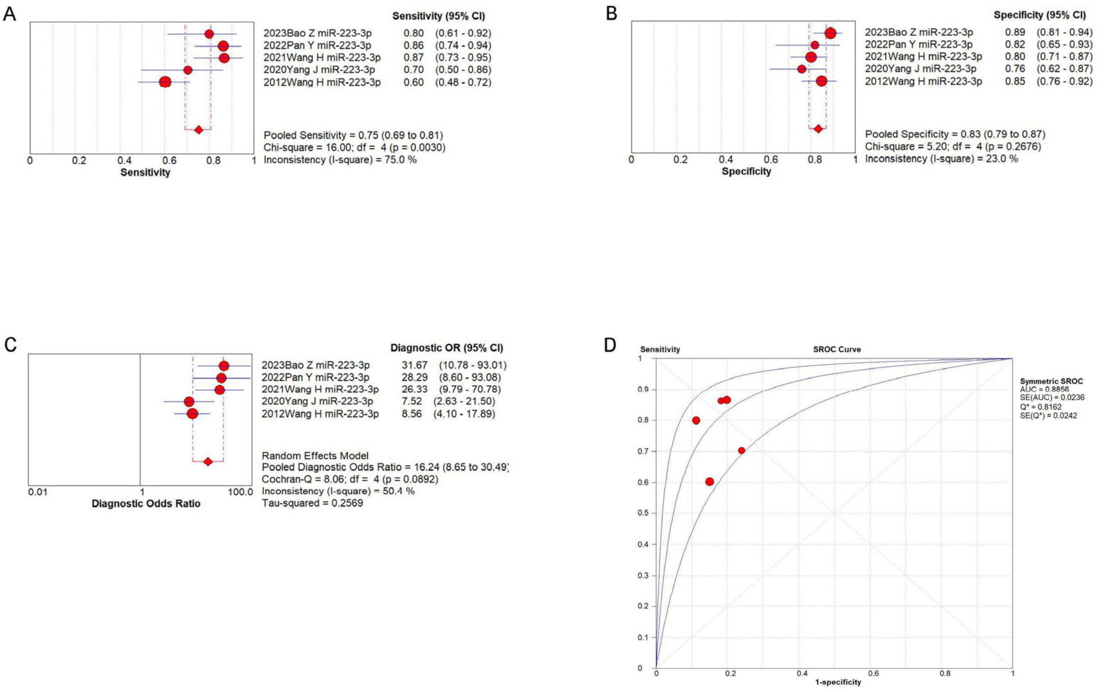

Supplementary Fig 8. Performance of miR-223-3p detection for sepsis diagnosis. (A) Pooled sensitivity. (B) Pooled specificity. (C) Overall DOR. (D) The SROCs for all datasets.

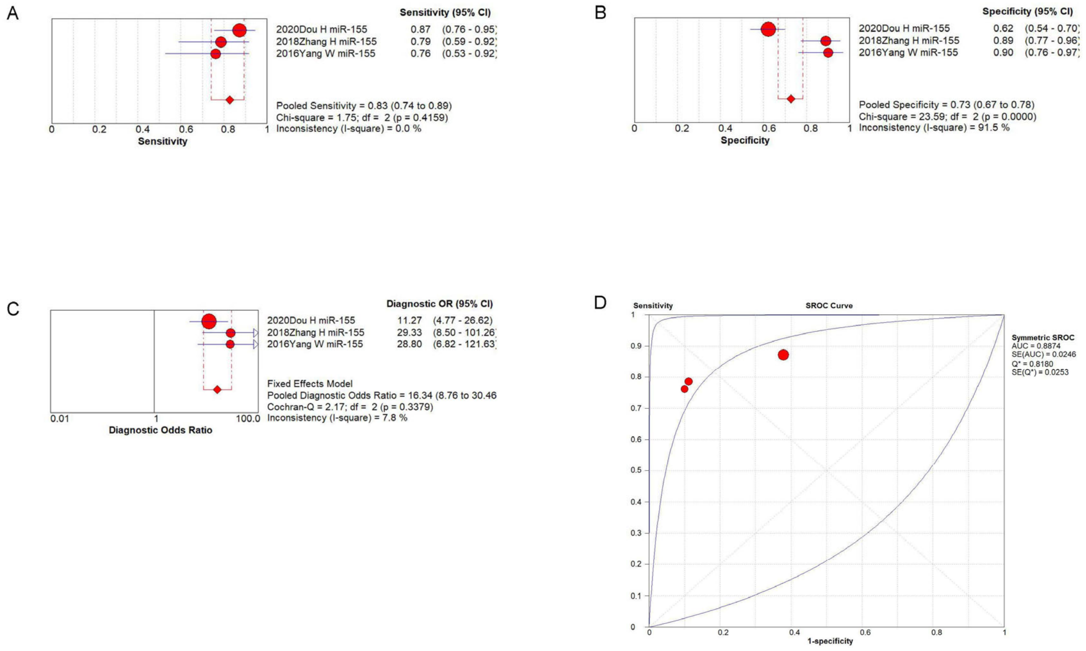

Supplementary Fig 9. Performance of miR-155 detection for sepsis diagnosis. (A) Pooled sensitivity. (B) Pooled specificity. (C) Overall DOR. (D) The SROCs for all datasets.

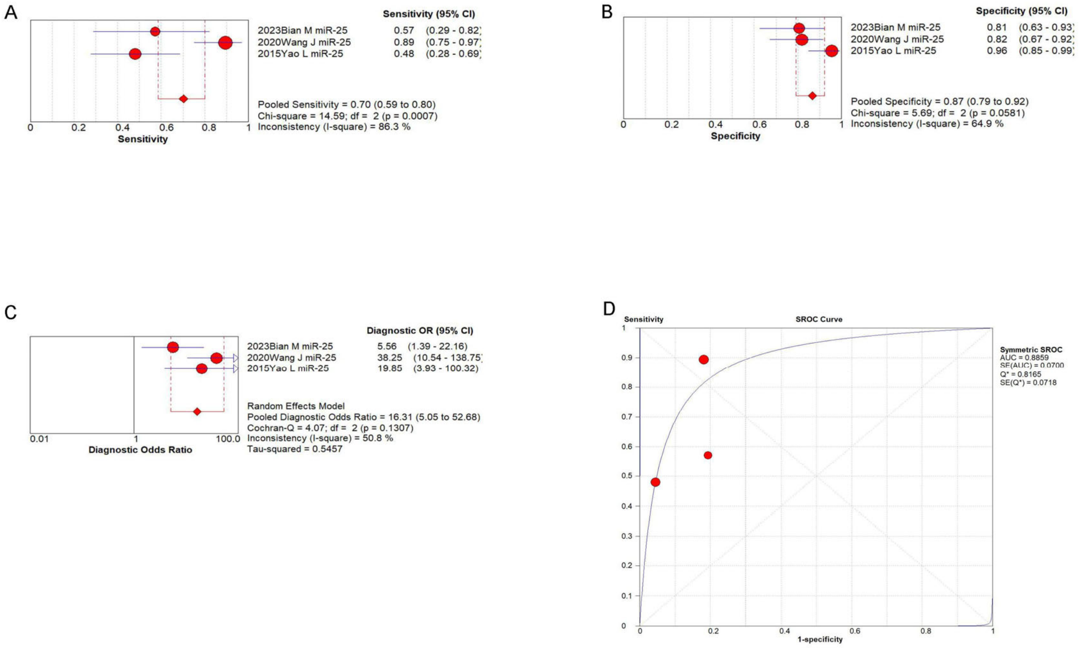

Supplementary Fig 10. Performance of miR-25 detection for sepsis diagnosis. (A) Pooled sensitivity. (B) Pooled specificity. (C) Overall DOR. (D) The SROCs for all datasets.

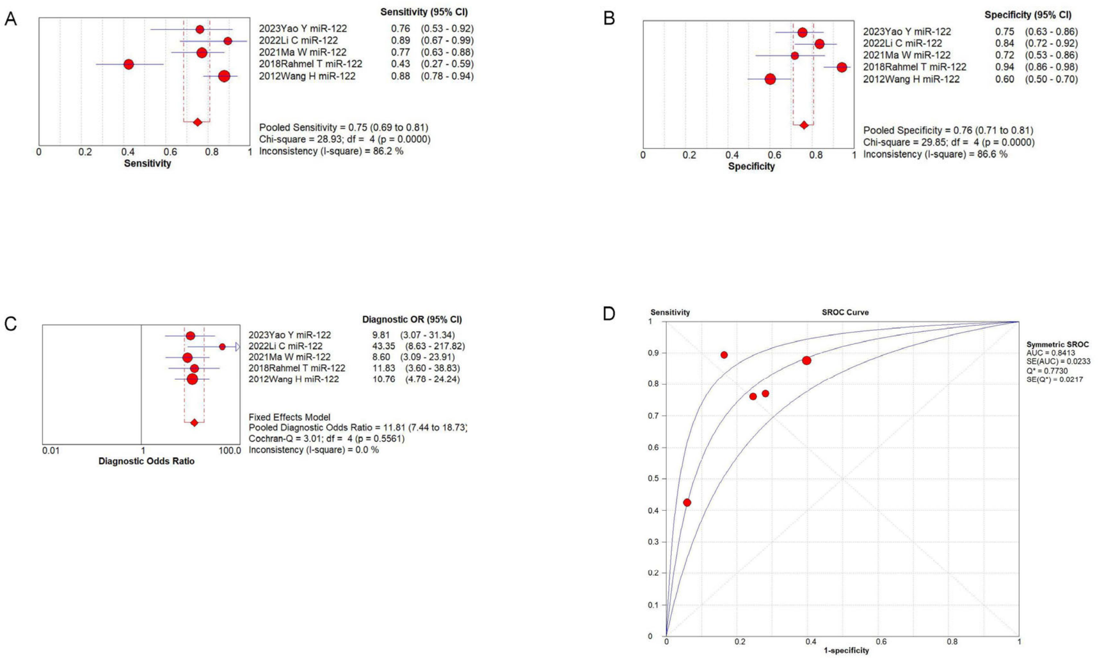

Supplementary Fig 11. Performance of miR-122 detection for sepsis diagnosis. (A) Pooled sensitivity. (B) Pooled specificity. (C) Overall DOR. (D) The SROCs for all datasets.

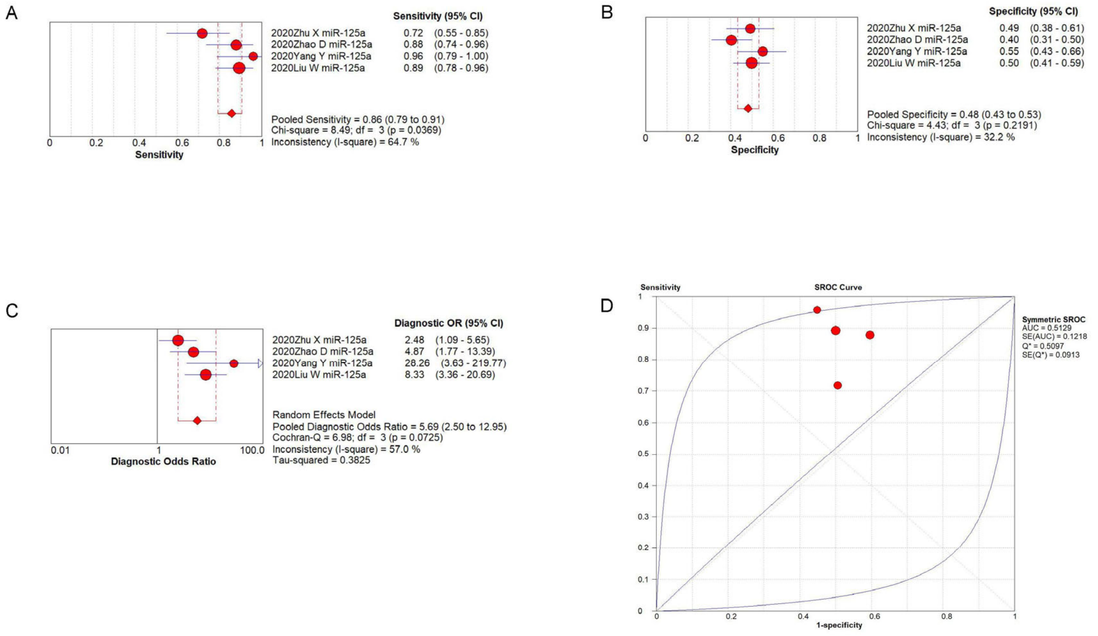

Supplementary Fig 12. Performance of miR-125a detection for sepsis diagnosis. (A) Pooled sensitivity. (B) Pooled specificity. (C) Overall DOR. (D) The SROCs for all datasets.

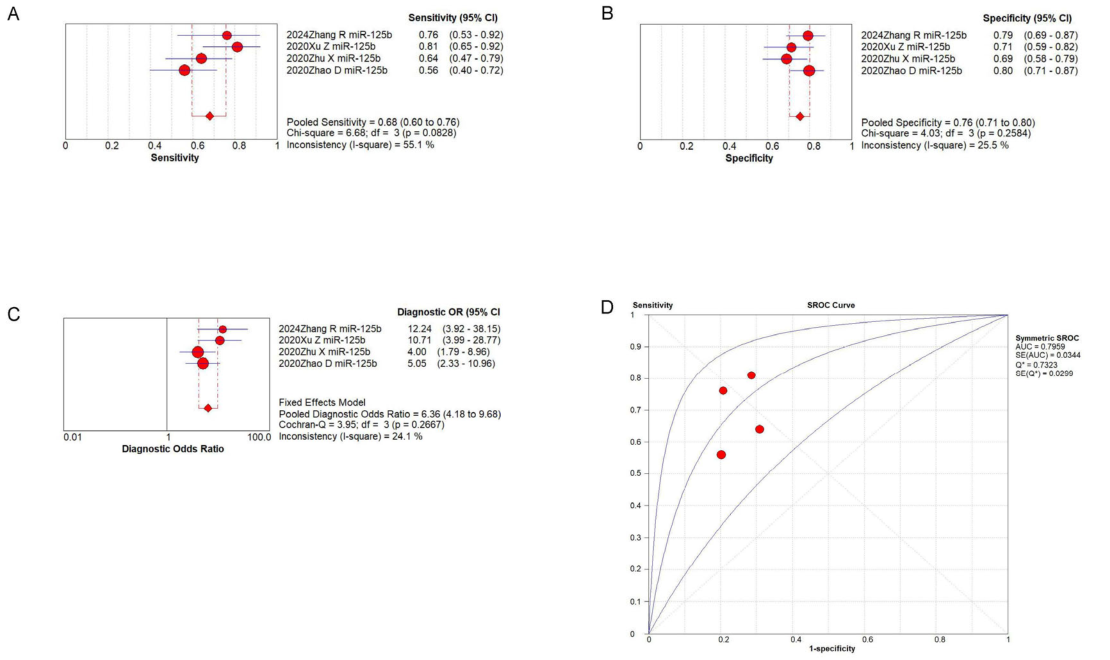

Supplementary Fig 13. Performance of miR-125b detection for sepsis diagnosis. (A) Pooled sensitivity. (B) Pooled specificity. (C) Overall DOR. (D) The SROCs for all datasets.

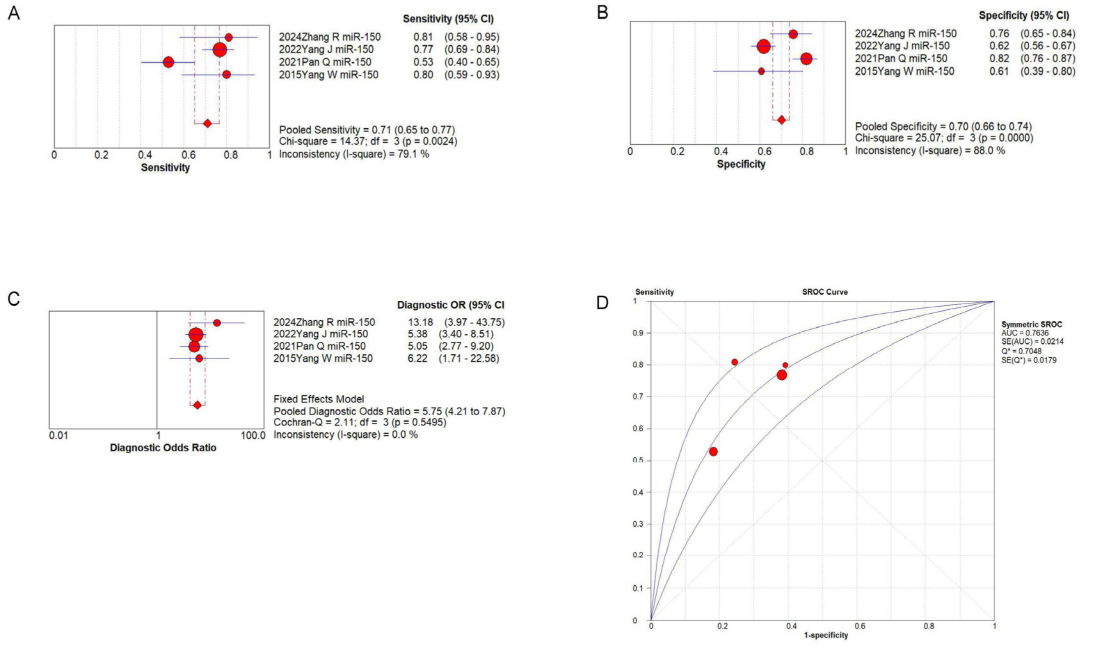

Supplementary Fig 14. Performance of miR-150 detection for sepsis diagnosis. (A) Pooled sensitivity. (B) Pooled specificity. (C) Overall DOR. (D) The SROCs for all datasets.
